# The microbiome-metabolome axis in colorectal cancer therapy resistance: mechanisms, mediators, and therapeutic opportunities

**DOI:** 10.3389/fonc.2026.1985675

**Published:** 2026-10-28

**Authors:** Anoud Khan, Aryan Tareen, Anusha Abdul Muqeet Farid, Ahrar Amin, Hamza Nawaz Khan, Danial Khan Hadi, Saqib Raza Khan, Afsheen Raza

**Affiliations:** 1 Department of Medicine, Ziauddin Medical College, Karachi, Pakistan; 2 Department of Medicine, Dow University of Health Sciences, Karachi, Pakistan; 3 Division of Medical Oncology, Department of Oncology, Schulich School of Medicine & Dentistry, Western University, London, ON, Canada; 4 Verspeeten Family Cancer Centre, London Health Sciences Centre, London, ON, Canada; 5 Department of Biomedical Sciences, College of Health Sciences, Abu Dhabi University, Abu Dhabi, United Arab Emirates; 6 Cancer Research Institute, Office of Research and Sponsored Programs, Abu Dhabi University, Abu Dhabi, United Arab Emirates

**Keywords:** chemotherapy, colorectal cancer, gut microbiota, immunotherapy, metabolome, microbial metabolites, microbiome, therapeutic resistance

## Abstract

Colorectal cancer (CRC) remains a leading cause of cancer-related morbidity and mortality worldwide, with therapeutic resistance representing a significant barrier to improved patient outcomes. Although tumor-intrinsic genetic alterations are important determinants of treatment resistance, emerging evidence suggests that the gut microbiome and its metabolites may also influence responses to chemotherapy, targeted therapy, and immunotherapy. The microbiome and metabolome function as an interconnected axis that can influence host immunity, microbial drug metabolism, epithelial homeostasis, and tumor-associated signaling pathways, thereby contributing to variation in therapeutic response. Despite advances in microbiome research, the mechanisms by which the microbiome-metabolome axis promotes therapy resistance are not fully elucidated, and clinical translation is limited by the absence of standardized biomarkers and validated therapeutic strategies. This review synthesizes current evidence on the molecular mechanisms through which the microbiome-metabolome axis contributes to CRC therapy resistance, with emphasis on microbial dysbiosis, bacterial metabolites, and host-microbial interactions in modulating treatment efficacy. Emerging microbiome-directed therapeutic approaches, including fecal microbiota transplantation, defined microbial consortia, dietary interventions, microbial metabolites, and engineered microbial therapies, are examined, along with the methodological and translational challenges that must be addressed before their integration into routine clinical practice.

## 1 Introduction

Colorectal cancer (CRC) constitutes a significant global health challenge and remains among the leading causes of cancer-related mortality. According to Global Cancer Statistics 2020 (GLOBOCAN), CRC accounted for approximately 1.9 million new cases and nearly 935,000 deaths, ranking as the third most commonly diagnosed cancer and the second leading cause of cancer-related mortality worldwide (1). Despite progress in screening and therapeutic interventions, CRC continues to exert a considerable burden, with recent United States-based surveillance data (SEER estimates) projecting over 150,000 new cases and approximately 50,000–53,000 deaths annually in 2025 (2).

Historically, CRC has been regarded as a disease predominantly affecting older adults, with most cases diagnosed after age 50, consistent with its slow, multistep carcinogenic process driven by cumulative genetic and environmental insults. However, recent data reveal a notable shift in disease distribution, characterized by a rising incidence of early-onset CRC (EOCRC) in individuals younger than 50 years (3, 4). This trend, observed alongside stable or declining incidence rates in older populations, indicates a genuine epidemiological transition rather than an artifact of enhanced screening. EOCRC now represents approximately 10% of all CRC cases and is often diagnosed at more advanced stages, resulting in poorer clinical outcomes compared to late-onset disease (5).

In addition to established risk factors, the gut microbiome is increasingly recognized as a potential mediator connecting environmental exposures to colorectal carcinogenesis. Lifestyle factors such as obesity, physical inactivity, and high consumption of processed foods, alcohol intake, antibiotic use, and low dietary fiber intake have been associated with reduction in beneficial commensals and an increase in pathobionts (5–7). These associations do not necessarily establish a direct causal relationship, as diet, host characteristics, medications, and other exposures may influence both microbial profiles and CRC risk. The consequences of dysbiosis extend beyond alterations in microbial composition, as gut microorganisms produce and modify a wide range of bioactive metabolites. Changes in metabolites such as short-chain fatty acids (SCFAs), secondary bile acids, and tryptophan-derived compounds can influence epithelial integrity, immune regulation, and oncogenic signaling within the colorectal microenvironment, although their effects depend on the metabolite, its concentration, and the biological context (6–8). Accordingly, the microbiome-metabolome axis provides a framework for investigating how microbial community composition and activity are associated with host metabolic outputs and downstream physiological processes (6, 8, 9).

Disruption of the microbiome-metabolome axis fosters a pro-tumorigenic environment characterized by chronic inflammation, compromised mucosal immunity, and metabolic reprogramming. Beyond facilitating tumor initiation and progression, microbial communities and their metabolites may also influence treatment response through immune modulation, microbial drug metabolism, and effects on tumor-cell survival and stress adaptation (10). In CRC, increased abundance of *Fusobacterium nucleatum* has been linked to reduced sensitivity to 5-fluorouracil and oxaliplatin, with experimental evidence implicating autophagy, apoptosis, and ferroptosis pathways, although the relative contribution of these mechanisms to treatment failure in patients remains incompletely established (11, 12). Additionally, gut microbial composition and metabolites have been associated with variability in immunotherapy response via modulation of antitumor immunity (12). Collectively, these findings indicate that microbial and metabolic interactions not only contribute to carcinogenesis but also actively modulate treatment efficacy, particularly as immunotherapy and precision oncology approaches expand in gastrointestinal malignancies (13, 14).

Despite increasing interest, comprehensive characterization of microbial composition and metabolite profiles in relation to treatment response remains limited. This gap impedes understanding of how microbial alterations translate into metabolic changes that affect tumor behavior, therapeutic efficacy, and resistance. Furthermore, significant inter individual and methodological variability in microbiome composition, along with key host-related confounders, complicates the identification of reproducible and clinically actionable microbial biomarkers (15).

Within this context, the present review synthesizes current evidence regarding the role of the microbiome-metabolome axis in CRC therapy resistance, with particular emphasis on molecular mechanisms, microbial mediators, metabolic pathways, and emerging therapeutic strategies aimed at overcoming resistance and improving clinical outcomes.

## 2 Materials and methods

A comprehensive literature search was conducted using PubMed/MEDLINE, Scopus, and Web of Science for peer-reviewed articles published from January 2000 to May 2026. Major search terms included: “Colorectal cancer,” “CRC,” “Microbiome,” “Metabolome,” “Therapy Resistance,” “Xenometabolism,” “beta-glucuronidase,” “SN-38,” “Cytidine deaminase,” “Pathobionts,” “*Fusobacterium nucleatum*,” “Colibactin,” “Postbiotics,” “Short-chain fatty acids (SCFAs),” “Inosine,” “STING activation,” “Synthetic biology,” “Living medicines,” and “Phage therapy.” Boolean operators (AND, OR, NOT), synonyms, alternative spellings, and Medical Subject Headings (MeSH) terms were incorporated to refine the search strategy. Only studies published in English were included to ensure consistency in result interpretation. Title and abstract screening preceded full-text quality assessment to determine relevance. Clinical trial data were verified with ClinicalTrials.gov to include the latest survival and response data for emerging consortia and engineered living medicines. Additional sources were identified through citation tracking and manual review of reference lists from landmark publications. After removing duplicates and studies lacking mechanistic data, 78 studies were included in the final analysis. Variability among individual studies was assessed to ensure a balanced evaluation of the current therapeutic landscape.

## 3 Gut microbiota and tumor microenvironment

CRC develops within a complex tumor ecosystem in which the gut microbiome, microbial metabolites, tumor cells, stromal components, and host immune cells continuously interact to influence tumor behavior and therapeutic response. The microbiome can therefore influence the tumor microenvironment (TME) through several interconnected mechanisms, including modulation of epithelial barrier integrity, inflammatory signaling, immune-cell recruitment, microbial metabolite production, and xenobiotic metabolism. These interactions provide a biological framework for investigating how microbial alterations may contribute to therapeutic resistance alongside tumor-intrinsic genetic mechanisms (9, 16–18).

In a healthy state, the gut microbiota maintains epithelial integrity, regulates immune tone, and produces metabolites that support controlled inflammation and cellular homeostasis. Microbial metabolites derived from dietary substrates such as SCFAs, bile acids, and tryptophan derivatives play a central role in maintaining the intestinal mucus barrier by regulating mucin (MUC2) expression, epithelial renewal, and antimicrobial defense (19). SCFAs, particularly butyrate, can contribute to epithelial energy metabolism and barrier integrity, whereas indole derivatives can activate host receptors including the aryl hydrocarbon receptor (AhR) and pregnane X receptor (PXR), thereby influencing epithelial and immune homeostasis (9, 16, 19, 20). In contrast, alterations in bile acid metabolism and microbial metabolite production can modify inflammatory and metabolic signaling within the intestinal environment (9, 16, 20).

This equilibrium is disrupted in CRC, leading to microbial dysbiosis characterized by reduced microbial diversity and enrichment of pathogenic species such as *F. nucleatum*, enterotoxigenic *Bacteroides fragilis (ETBF), Streptococcus gallolyticus* and colibactin-producing *Escherichia coli* (21–24). However, the presence of these organisms should not automatically be interpreted as evidence of causality, because microbial composition is strongly influenced by host characteristics, diet, treatment exposure, bowel transit, and other clinical factors (15, 25). Several of these organisms nevertheless possess experimentally demonstrated mechanisms capable of influencing tumor biology, including modulation of epithelial signaling, DNA damage, inflammatory pathways, and immune therapeutic response (26).

The mechanisms proposed to link dysbiosis to resistance extend beyond changes in microbial composition. In experimental models, microbial signaling has been shown to activate pro-survival pathways, including NF-κB and STAT3, and to enhance autophagy, suppress apoptosis, and alter drug metabolism, processes that may reduce tumor susceptibility to chemotherapy (11, 27). Similarly, the microbiome may also influence immunotherapy by modifying antigen presentation, T-cell activation, inflammatory tone, and the availability of metabolites that affect immune-cell function (28). These effects are particularly relevant to microsatellite-stable (MSS) or mismatch-repair-proficient (pMMR) CRC, in which immune exclusion and an immunosuppressive TME contribute to limited responsiveness to immune checkpoint inhibitors (ICIs) (29, 30). Experimental and observational evidence implicates regulatory T cells and myeloid-derived suppressor cells in immunosuppressive responses, alongside reduced cytotoxic CD8^+^ T-cell activity; however, the extent to which particular microbial alterations cause these changes in patients with CRC remains to be established (31–33).

These findings prompt consideration of whether microbiome and metabolome profiling could become part of routine oncologic assessment. If specific microbial signatures or metabolite patterns reliably predict resistance, they may be used to stratify patients and guide therapy selection. Integrated multi-omics approaches combining tumor genomics, microbial profiles, metabolite measurements, and relevant clinical variables may improve the characterization of factors associated with tumor behavior and therapeutic response. However, microbiome and metabolome profiling remain investigational for routine CRC treatment selection, and their clinical utility will depend on prospective validation and demonstration of added value beyond established biomarkers (**Figure 1**).

**Figure 1:**
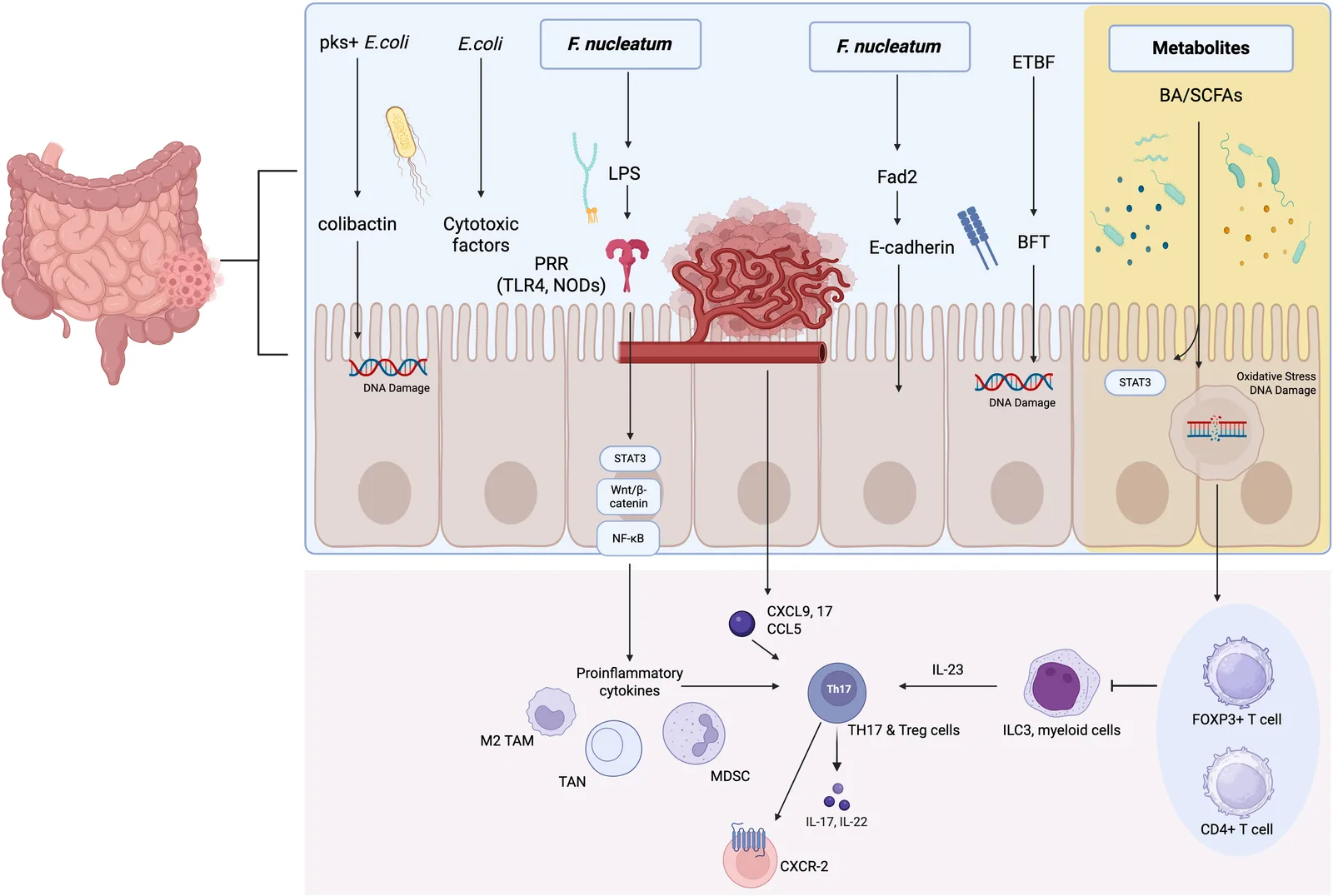
Gut microbiome dysbiosis promotes colorectal cancer progression and therapy resistance. A healthy intestinal microbiota maintains epithelial barrier integrity, immune homeostasis, and balanced microbial metabolism. Dysbiosis promotes expansion of pathogenic bacteria, disruption of the intestinal barrier, microbial toxin production, chronic inflammation, and metabolic alterations that remodel the tumor microenvironment. These changes activate oncogenic signaling pathways, promote immune evasion, and contribute to resistance against chemotherapy, immunotherapy, and radiotherapy.

### 3.1 Mechanistic insights into therapy resistance

#### 3.1.1 Inflammatory survival signaling

The microbiome-metabolome axis is proposed to contribute to CRC therapy resistance through interconnected inflammatory, immune, and metabolic mechanisms that collectively reshape the TME and enhance tumor adaptation to therapeutic stress (11, 17, 18). This is particularly relevant in CRC because many standard therapies, including fluoropyrimidines, platinum agents, irinotecan, radiotherapy, targeted therapy, and immune checkpoint inhibitors, ultimately depend on the induction of DNA damage, oxidative stress, apoptosis, and immune-mediated tumor clearance (34–36). Preclinical evidence suggests that microbiota-driven signaling may produce intrinsic resistance by pre-conditioning tumor cells toward anti-apoptotic and stem-like states before treatment exposure, while chronic exposure to microbial and metabolic stimuli during therapy can drive acquired resistance through selection of tolerant subclones, induction of protective autophagy, epigenetic reprogramming of drug-response genes, and remodeling of the immune microenvironment away from cytotoxic surveillance. In this sense, the microbiome-metabolome axis may function less as a single discrete resistance mechanism and more as a modifiable ecological layer that determines how readily tumor cells can access pre-existing survival and adaptation programs under therapeutic pressure (37, 38).

A central mechanism involves chronic inflammation driven by microbial dysbiosis. Disruption of epithelial barrier integrity increases the translocation of microbial products, such as lipopolysaccharide, peptidoglycans, and bacterial DNA, into the TME, where they activate pattern-recognition receptors, including Toll-like receptors and NOD-like receptors, on epithelial and immune cells (18, 39, 40). This signaling converges on NF-κB and STAT3, transcription factors whose target genes overlap extensively and include the anti-apoptotic and pro-proliferative mediators BCL-2, BCL-XL, survivin, and cyclin D1 (41, 42). By upregulating these targets, microbial signaling may counteract the apoptotic and cell-cycle arrest programs that chemotherapy, radiotherapy, and other cytotoxic therapies depend on, which could reduce tumor susceptibility to treatment-induced cell death (43–46). Persistent activation of this axis may additionally promote oxidative stress, genomic instability, and the emergence of resistant tumor subclones under therapeutic pressure (47). Bacterial products, cytokines, and metabolites converge on this same limited set of NF-κB/STAT3 target genes, so a therapy-resistant phenotype can emerge even when no single microbial species or signaling pathway predominates, which may explain why similar resistance phenotypes recur across patients with heterogeneous microbiota profiles. However, this remains a mechanistic hypothesis that requires direct validation in patient cohorts.

A well-studied example from experimental models is ETBF, which secretes *B. fragilis* toxin, disrupts epithelial tight junctions, and induces IL-17-mediated inflammation (48). IL-17-driven inflammation sustains neutrophil recruitment and STAT3 activation, promoting tumor progression and survival signaling (48, 49). Beyond ETBF, other bacteria-associated inflammatory pathways also converge on the IL-6/STAT3 and NF-κB signaling pathways. Tumor-infiltrating leukocytes isolated from CRC tissue secrete IL-6 and TNF-α, which act synergistically to activate STAT3 and NF-κB in tumor cells and promote tumor cell proliferation and survival (50). IL-6 has also been shown to promote colorectal cancer stem-like properties through the STAT3-FRA1-NANOG axis, with inhibition of this pathway reducing chemoresistance, migration, and sphere-forming capacity, providing a direct link between IL-6 signaling and treatment resistance (51). In addition, microbiota-dependent IL-23 production by myeloid cells, triggered by translocation of microbial products across a disrupted epithelial barrier, promotes expansion of IL-17-producing T cells and accelerates adenoma-to-carcinoma progression in preclinical models (52). Clinical studies have also reported elevated IL-1β, IL-6, and TNF-α in patients with CRC, with higher levels associated with more advanced disease and other markers of inflammation, further supporting the presence of an interconnected inflammatory cytokine network in CRC (53) (**Figure 2**).

**Figure 2:**
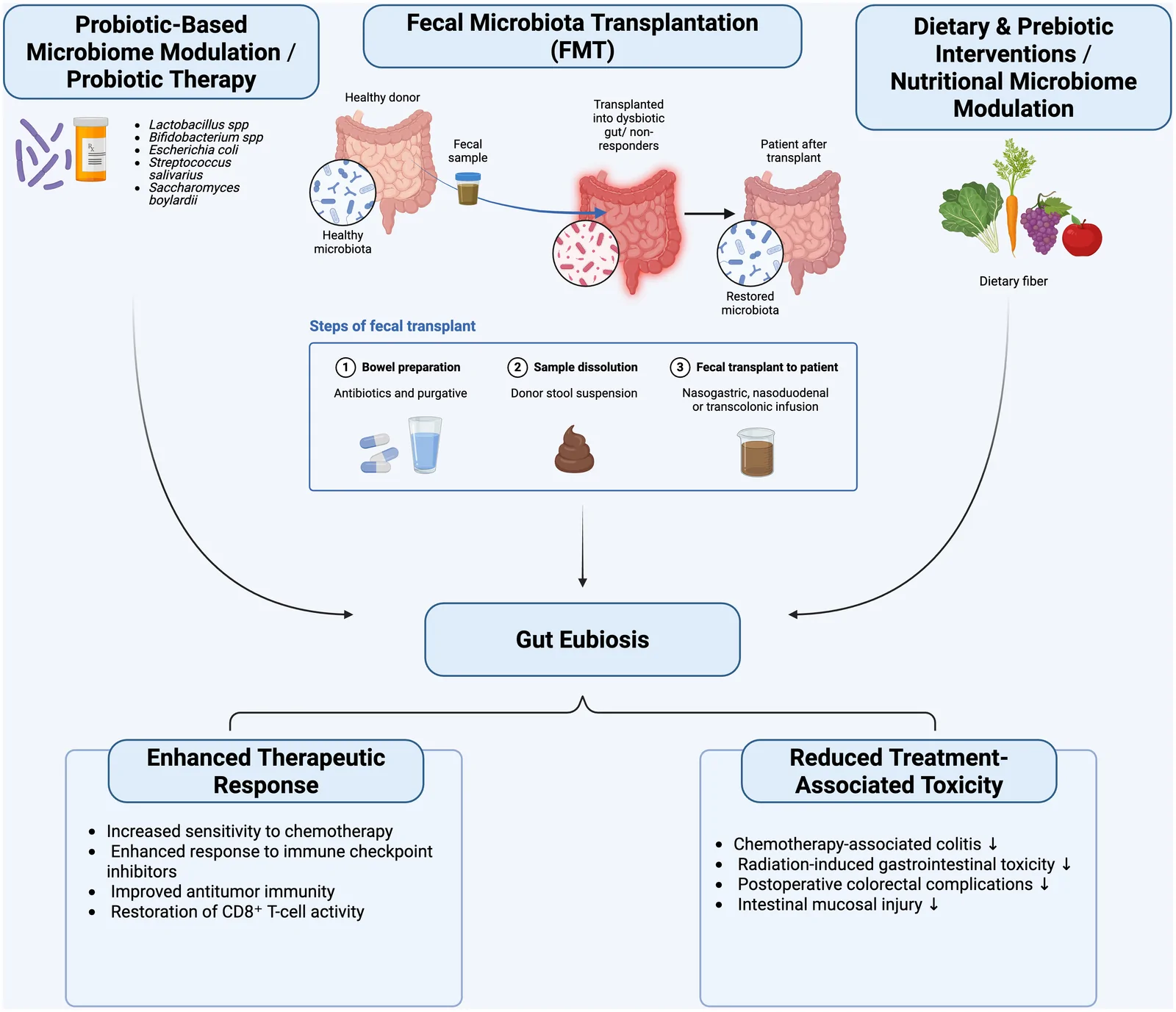
Molecular mechanisms by which the gut microbiome promotes therapy resistance in colorectal cancer. Representative pathogenic bacteria, including *pks^+^ Escherichia coli*, *Fusobacterium nucleatum*, and enterotoxigenic *Bacteroides fragilis*, induce DNA damage, epithelial barrier dysfunction, inflammatory signaling, and immune remodeling through activation of STAT3, NF-κB, Wnt/β-catenin, and related pathways. Altered microbial metabolites further promote tumor progression and immune evasion, collectively driving resistance to anticancer therapy.

#### 3.1.2 Autophagy and stress adaptation

Beyond inflammatory transcriptional activation, experimental studies indicate that CRC-associated bacteria can reprogram tumor stress-response pathways and may promote chemotherapy resistance (11, 54). Among the most extensively studied organisms is *F. nucleatum*, which is enriched in CRC tissue and has been associated with advanced disease and poorer outcomes (11, 55). In CRC cell lines and xenograft models, *F. nucleatum* was shown to promote resistance to fluoropyrimidines and platinum-based chemotherapy by inducing protective autophagy through downregulation of miR-18a and miR-4802, leading to increased expression of ULK1 and ATG7. Enhanced autophagic activity limits apoptosis and enables tumor cells to survive exposure to 5-fluorouracil and oxaliplatin (11). This mechanism has also been associated with CRC recurrence, with recurrent tumors showing higher *F. nucleatum*, ULK1, and ATG7 expression and lower miR-18a/miR-4802 levels (11, 56).

Autophagy-mediated resistance is not restricted to *F. nucleatum*. In CRC models, *Porphyromonas gingivalis* infection induces autophagic degradation of STING, reducing cGAS–STING-dependent antitumor immune signaling and potentially contributing to immune evasion (57). Evidence from other gastrointestinal cancers further suggests that *P. gingivalis* can alter autophagy through a YAP/TAZ–miR-21-5p–ERK pathway, promoting malignant progression and chemoresistance (58, 59), although its significance in CRC remains less established. Together, these findings suggest that bacteria can promote treatment resistance by activating autophagy, although the upstream pathways differ across bacterial species.

#### 3.1.3 Metabolic and epigenetic reprogramming

Microbial metabolites represent a major functional interface through which the microbiome influences CRC behaviour and therapeutic response. Dysbiosis can alter the production of key metabolites, including SCFAs, secondary bile acids, polyamines, and tryptophan-derived indoles, which can influence tumor metabolism, immune signaling, and epigenetic regulation (60). CRC is also associated with alterations in SCFA-producing bacteria, including *Faecalibacterium prausnitzii*, and broader changes in microbial metabolite profiles that may disrupt epithelial and immune homeostasis, although altered abundance alone does not establish a functional effect on treatment response (61).

Polyamines such as putrescine, spermidine, and spermine support tumor proliferation and cellular stress adaptation and are frequently dysregulated in CRC (62). Oxidative stress contributes to the cytotoxic effects of several CRC treatments, and microbiome-associated metabolic changes may influence the balance between ROS generation and antioxidant defenses and thereby affect treatment response (63, 64). However, although polyamine metabolism can modulate oxidative and metabolic stress, its direct contribution to chemotherapy resistance in CRC remains less well established than that of other microbiota-derived metabolites, and further evidence is needed to define its role in treatment resistance.

Tryptophan-derived microbial metabolites can also modify antitumor immunity through the aryl hydrocarbon receptor (AhR). Importantly, these effects appear to be metabolite- and context-dependent rather than uniformly immunosuppressive. For example, indole-3-propionic acid has been reported to enhance CD8^+^ T-cell infiltration and improve 5-fluorouracil sensitivity through AhR signaling in preclinical CRC models (65–67). This finding supports the need to evaluate individual metabolites and signaling pathways rather than treating all tryptophan-derived compounds as functionally equivalent.

Bile-acid metabolism provides another potential mechanism of treatment resistance. Secondary bile acids can activate survival pathways including PI3K/AKT and NF-κB in colorectal epithelial and cancer cells, promoting proliferation and resistance to apoptosis (65, 68). More recent evidence also links altered bile-acid signaling to immunotherapy response. Separately, CRC-model evidence has linked glycolic acid-associated signaling through an FXR-SOX14-DHHC9 axis to increased PD-L1 expression and impaired CD8^+^ T-cell activity, and targeting this pathway enhanced anti-PD-1 activity in the reported models (69). These findings are mechanistically informative but do not establish that a particular bile-acid profile predicts treatment response in patients. Prospective clinical studies are needed to determine which metabolite signatures are reproducible, whether they add predictive value beyond established clinical and molecular factors, and whether modifying them can safely improve treatment outcomes.

The microbiome-metabolome axis may also influence therapeutic response through epigenetic mechanisms. SCFAs, particularly butyrate, provide the clearest example of microbiome-mediated epigenetic regulation. Butyrate acts as a histone deacetylase inhibitor and has generally protective effects in normal colonic epithelium. However, its effects in CRC are influenced by tumor metabolic state, contributing to the well-described “butyrate paradox.” CRC cells often oxidize less butyrate than normal colonocytes, allowing greater intracellular accumulation and HDAC inhibition (65, 70). Importantly, chronic butyrate exposure can induce acquired butyrate resistance in CRC cell models through protective autophagy. Butyrate-resistant cells showed increased Beclin-1 and LC3B-II expression, with reduced AMPK/ACC signaling and activation of Akt/mTOR; pharmacologic activation of AMPK restored butyrate sensitivity, supporting a role for AMPK/Akt/mTOR-regulated autophagy in this adaptive response (71). Conversely, butyrate can enhance ferroptosis through epigenetic regulation of xCT. By inhibiting class I HDAC activity, butyrate increases c-Fos expression, suppresses xCT-dependent glutathione synthesis, and sensitizes CRC cells to ferroptosis. Experimental studies further show that butyrate enhances the pro-ferroptotic activity of oxaliplatin, while clinical specimens demonstrate higher xCT expression in oxaliplatin non-responders than in responders (72). Overall, the effect of microbial metabolites on treatment response is highly context-dependent, with the same metabolite potentially exerting different effects depending on tumor metabolism, exposure, and the cellular pathway involved.

#### 3.1.4 EMT-driven drug resistance and cellular plasticity

The inflammatory and metabolic alterations described above ultimately converge on epithelial-to-mesenchymal transition (EMT), an important contributor to tumor plasticity and therapeutic resistance. Activation of NF-κB, STAT3, and Wnt/β-catenin signaling increases expression of EMT-associated transcription factors, including SNAIL, TWIST, and ZEB1, promoting invasive behaviour, resistance to apoptosis, and reduced sensitivity to chemotherapy (73). EMT-associated states have also been linked to increased drug efflux and persistence of more therapy-tolerant tumor cell populations (74).

Experimental studies suggest that the microbiome can contribute to this process. ETBF and its toxin, BFT, cleave E-cadherin and activate β-catenin and NF-κB signaling, while ETBF/BFT also activate the STAT3-ZEB2 axis and promote EMT in CRC models. BFT-mediated E-cadherin cleavage therefore provides a direct link between microbial exposure, epithelial disruption, and pro-tumor signaling (75). Whether this contributes to therapy resistance in patients has not been established.

Autophagy and β-catenin signaling (Section 3.1.2) can further reinforce EMT-associated chemoresistance and stemness, as shown for the SOX2–β-catenin/Beclin-1 axis (76, 77), indicating overlapping rather than separate resistance pathways.

Post-transcriptional regulation provides an additional layer of control. The untranslated regions of SNAIL and ZEB1 participate in a competing endogenous RNA network involving tumor-suppressive miR-34a and miR-200. Disruption of this network increased EMT-related phenotypes and paclitaxel resistance in CRC cells, indicating that EMT-associated resistance can also be maintained through RNA-level regulation (73). Together, these changes may contribute to relapse with more aggressive disease despite an initial treatment response.

#### 3.1.5 Immune and pharmacologic modulation

In addition to regulating tumor biology directly, the microbiome influences therapeutic efficacy through immune modulation and alterations in drug pharmacology. Dysbiosis can promote expansion of regulatory T cells, myeloid-derived suppressor cells, and M2-like macrophages, which impair CD8+ T-cell function through IL-10, TGF-β signaling, arginase activity, and reactive nitrogen species (78). Microbial metabolites may further reinforce immune exclusion, contributing to the limited efficacy of checkpoint inhibitors in microsatellite-stable CRC (78).

*F. nucleatum* inhibits antitumor immune activity through its Fap2 adhesin, which binds the inhibitory receptor TIGIT on NK and T cells (79). *Peptostreptococcus anaerobius* provides another mechanism: it activates integrin α2β1–NF-κB signaling in CRC cells to induce CXCL1 and recruit CXCR2+ MDSCs, while also directly increasing MDSC suppressive activity through Slamf4-dependent ARG1 and iNOS expression. In mouse models, these effects reduced the response to anti-PD-1 therapy (80).

Microbial metabolites can further alter immune-cell trafficking. *F. nucleatum*-derived succinic acid activates the SUCNR1–HIF-1α–EZH2 axis, suppressing cGAS–IFN-β signaling and reducing CCL5 and CXCL10 expression, which limits CD8+ T-cell recruitment and weakens the response to PD-1 blockade in CRC models (81). Similarly, the microbial metabolite 4-hydroxybenzeneacetic acid (4-HPA) activates JAK2/STAT3 signaling in CRC cells, increasing CXCL3 production and recruitment of PMN-MDSCs. Inhibition of CXCL3 or reduction of 4-HPA improved the response to anti-PD-1 therapy in preclinical models (82).

Tumor-intrinsic pathways can further reinforce immune exclusion. Activation of Wnt/β-catenin signaling in CRC is associated with reduced CD8+ T-cell infiltration and impaired antitumor immunity, providing a parallel mechanism through which tumor cells can limit checkpoint blockade efficacy (83). Conversely, specific commensals, including *Bifidobacterium, Bacteroides*, and *Akkermansia*, can enhance dendritic-cell and effector T-cell responses and improve checkpoint inhibitor activity in preclinical models (84–86). Similar associations between *Akkermansia* and improved PD-1 blockade response have also been reported in non-CRC clinical cohorts (85). However, their therapeutic benefit in CRC remains largely preclinical and requires clinical validation.

The microbiome also influences drug exposure through metabolic reprogramming. Irinotecan is converted to the active metabolite SN-38 and subsequently glucuronidated to inactive SN-38G, which is excreted into the intestinal lumen. Bacterial β-glucuronidases can reconvert SN-38G to SN-38, causing intestinal injury and diarrhea (87). Although primarily a toxicity mechanism, this often leads to dose reductions or treatment interruption, reducing relative dose intensity and potentially compromising efficacy (87, 88). Collectively, these largely preclinical findings suggest that resistance may arise from a dynamic host-microbiome ecosystem in addition to tumor-intrinsic factors.

### 3.2 Clinical implications

#### 3.2.1 Microbiome-metabolome profiling in CRC

Standard CRC workup includes tumor molecular profiling (KRAS, MSI, BRAF). Minimally invasive biomarkers such as circulating tumor DNA are also being incorporated into precision oncology (89, 90). Emerging evidence supports the potential value of integrating microbiome and metabolome profiling into precision oncology, as baseline microbial and metabolic features have been associated with treatment response and resistance in CRC in some cohorts, but the strength and clinical relevance of these associations vary across studies (91–93). Microbial metabolites detectable in stool, plasma, and urine offer a non-invasive means of interrogating the TME in real time. Characteristic alterations, including depleted SCFAs, elevated secondary bile acids, and altered tryptophan catabolites, have been consistently associated with diminished immunotherapy efficacy and chemotherapy resistance in CRC patients, but no universal metabolite signature has been established for predicting resistance in routine practice. The principal clinical utility of such profiling lies in its pre-treatment use, enabling resistance prediction and patient stratification prior to therapeutic commitment (94). The integration of metagenomic sequencing with metabolomic profiling yields meaningfully improved sensitivity for resistance prediction and patient risk stratification compared to either modality used in isolation, supporting their combined use during pre-treatment assessment (95). Patients who subsequently develop chemotherapy resistance have also been shown to exhibit distinct baseline microbial profiles, suggesting that microbiome profiling could help identify high-risk patients and inform more individualized therapeutic decisions (96).

However, the clinical translation of microbiome-metabolome profiling remains limited by substantial heterogeneity across cohorts and methodologies. Microbial signatures are influenced by diet, medications, geography, bowel transit, treatment exposure, and analytical methodology (12, 97, 98), while differences in sample collection, storage, DNA extraction, sequencing platforms, taxonomic databases, metabolomic platforms, and bioinformatic pipelines can further affect microbial and metabolic profiles (12, 97). The 2024 Nature Medicine study by Tito et al. demonstrated that adjustment for clinical and technical confounders can substantially alter apparently robust CRC-associated microbial signals, emphasizing the need for rigorous and standardized profiling (15). Antibiotic exposure represents an additional clinically relevant confounder, particularly in patients receiving immunotherapy, as antibiotics can disrupt commensal bacteria associated with ICI efficacy. A meta-analysis has associated antibiotic use around ICI treatment with poorer progression-free and overall survival, highlighting the need to account for antibiotic exposure when interpreting microbiome-based biomarkers and designing prospective trials (99).

Accordingly, these approaches should currently be considered investigational rather than clinically validated biomarkers. Before routine implementation, candidate microbial and metabolic signatures require prospective validation across geographically and clinically diverse populations, standardized analytical workflows, predefined diagnostic thresholds, and demonstration of incremental predictive value beyond established clinicopathological and tumor-derived biomarkers. Metagenomic sequencing can provide information on microbial functional capacity beyond taxonomic composition, while integration with tumor genomics, immune profiling, and clinical variables may ultimately improve prediction of therapeutic response. Early multi-omics machine-learning studies support the feasibility of this approach, but their clinical utility remains dependent on external validation and prospective assessment against clinically meaningful outcomes (100).

#### 3.2.2 Microbiota-mediated chemotherapy resistance

The efficacy of standard chemotherapy regimens in CRC is not determined by tumor genetics alone. Specific gut bacteria actively interfere with drug function through defined molecular mechanisms, with direct consequences for treatment outcomes. Among the most clinically significant of these interactions is the enzymatic inactivation of 5-fluorouracil (5-FU) by gut bacteria. *E. coli* encodes enzymes from the preTA operon that metabolize 5-FU to the less active metabolite dihydrofluorouracil (DHFU), providing a potential microbiome-mediated mechanism for reducing fluoropyrimidine activity (101, 102). In experimental models, *F. nucleatum* induces protective autophagy that reduces chemotherapy sensitivity (11). These findings suggest that microbial abundance and functional capacity may contribute to interindividual variation in chemotherapy exposure and cellular drug sensitivity, although the extent to which these mechanisms alter intratumoral drug concentrations in patients remains incompletely defined (102). Similarly, *B. fragilis* contributes to broader multidrug resistance through SusD/RagB-dependent activation of Notch1; this organism triggers epithelial-mesenchymal transition and cancer stem cell acquisition in CRC cells, rendering them resistant not to a single agent but to multiple chemotherapeutic drugs simultaneously (103).

Irinotecan-related gastrointestinal toxicity represents another microbiome-mediated phenomenon with immediate clinical relevance. Selective β-glucuronidase inhibitors that spare the wider microbiota are discussed in Section 3.3.1 (75). Conversely, microbial metabolites can also enhance chemotherapy efficacy. Sodium butyrate, a SCFA produced by fiber-fermenting bacteria, has been shown to synergize with oxaliplatin by promoting apoptosis and suppressing proliferation, migration, and invasion in CRC cells (104). Restoring butyrate-producing microbiota through dietary modification or prebiotic supplementation may therefore represent a potential strategy to sensitize tumors to standard chemotherapy, as increased butyrate production may enhance apoptosis, inhibit tumor-cell proliferation, and modulate inflammatory and immune signaling within the TME (104, 105). However, clinical evidence supporting these approaches for improving chemotherapy response remains limited, and their efficacy and safety require prospective validation.

#### 3.2.3 The microbiome as a determinant of immunotherapy response

Beyond tumor-intrinsic determinants such as microsatellite instability status, the gut microbiome has emerged as an independent and potentially modifiable predictor of immunotherapy response. Pre-treatment gut microbiome composition has been shown to correlate significantly with clinical response to PD-1 and PD-L1 inhibitors in patients with CRC. Higher microbial diversity, alongside enrichment of specific taxa including *Akkermansia muciniphila, Bifidobacterium*, and *F. prausnitzii*, has been associated with improved progression-free and overall survival in patients receiving immune checkpoint inhibitor therapy (106).

At the metabolite level, tryptophan-derived microbial metabolites, particularly indole-3-propionic acid and indole-3-acetic acid, have been shown to augment CD8+ T cell infiltration and cytokine secretion within the TME (100). These metabolites may enhance antitumor immunity by promoting T-cell activation and cytokine production while influencing tumor-cell stemness and epithelial-mesenchymal transition (100); however, the extent to which these effects translate into improved ICI efficacy in CRC remains uncertain.

The clinical urgency of this issue is particularly pronounced in microsatellite-stable CRC, which accounts for most metastatic cases and derives virtually no benefit from currently approved checkpoint inhibitors (107). For these patients, microbiome modulation is one of the few mechanistically grounded strategies that could convert a non-responsive tumor environment into a responsive one, although this remains an area of active investigation (29). Early-phase clinical studies in melanoma have demonstrated that fecal microbiota transplantation (FMT) from immunotherapy-responsive donors can restore sensitivity to PD-1 inhibitors in previously refractory patients. This evidence is currently strongest in melanoma patients, with subsequent studies extending the approach to other solid tumors (108–111). For example, responder-derived FMT combined with pembrolizumab produced clinical responses in a subset of patients with anti-PD-1-refractory melanoma (109, 110). More recently, the phase II FMT-LUMINate trial evaluated first-line FMT plus immune checkpoint blockade in melanoma and non-small cell lung cancer and reported an objective response rate of 80% in the NSCLC cohort, providing further proof-of-concept that microbiome remodeling can influence immunotherapy efficacy (111). However, these findings should not be directly extrapolated to CRC, where prospective clinical evidence remains limited, underscoring the need for CRC-specific clinical trials evaluating FMT and other microbiome-directed strategies.

#### 3.2.4 Multi-omics integration and the path to precision oncology

The heterogeneity of CRC treatment outcomes cannot be fully explained by tumor genomics alone. Integrating microbiome and metabolome data with host genomic and immune profiling offers a more comprehensive framework for individualized treatment prediction. Next-generation sequencing of the gut microbiome, combined with metabolomic profiling and analyzed through machine learning algorithms, has demonstrated the capacity to identify resistance-associated microbial signatures with greater predictive accuracy than any single data modality.

Patient-derived organoids offer a complementary and highly individualized platform for translating this data into treatment decisions. Grown directly from a patient’s own tumor tissue and exposed to their specific microbial and metabolite environment, organoids enable drug-sensitivity testing that accounts for each patient’s unique microbial context. Combined with spatial transcriptomics and proteomic profiling, this approach enables individual-level stratification of metabolic vulnerabilities, shifting drug selection from probability to precision (95).

The envisioned goal is a pre-treatment multi-omic dashboard that integrates tumor genomics, gut microbiome composition, and metabolite profiles to generate individualized predictions of resistance and inform therapeutic decisions prior to therapy initiation. Realizing this objective will require advances in clinical infrastructure, prospective validation, and regulatory frameworks that recognize the microbiome as an integral component of precision oncology.

### 3.3 Therapeutic opportunities

The recognition of the microbiome-metabolome axis as a critical determinant of therapeutic success in CRC has catalyzed a paradigm shift from observational oncology to interventional “metabolic bioremediation.” Rather than focusing solely on tumor-intrinsic mechanisms of resistance, emerging multi-omics studies highlight the therapeutic potential of targeting microbial metabolism, bacterial virulence factors, and host-microbiome interactions to restore drug sensitivity, mitigate treatment-related toxicities, and overcome the immunosuppressive barriers characteristic of CRC (112) (**Figure 3**).

**Figure 3:**
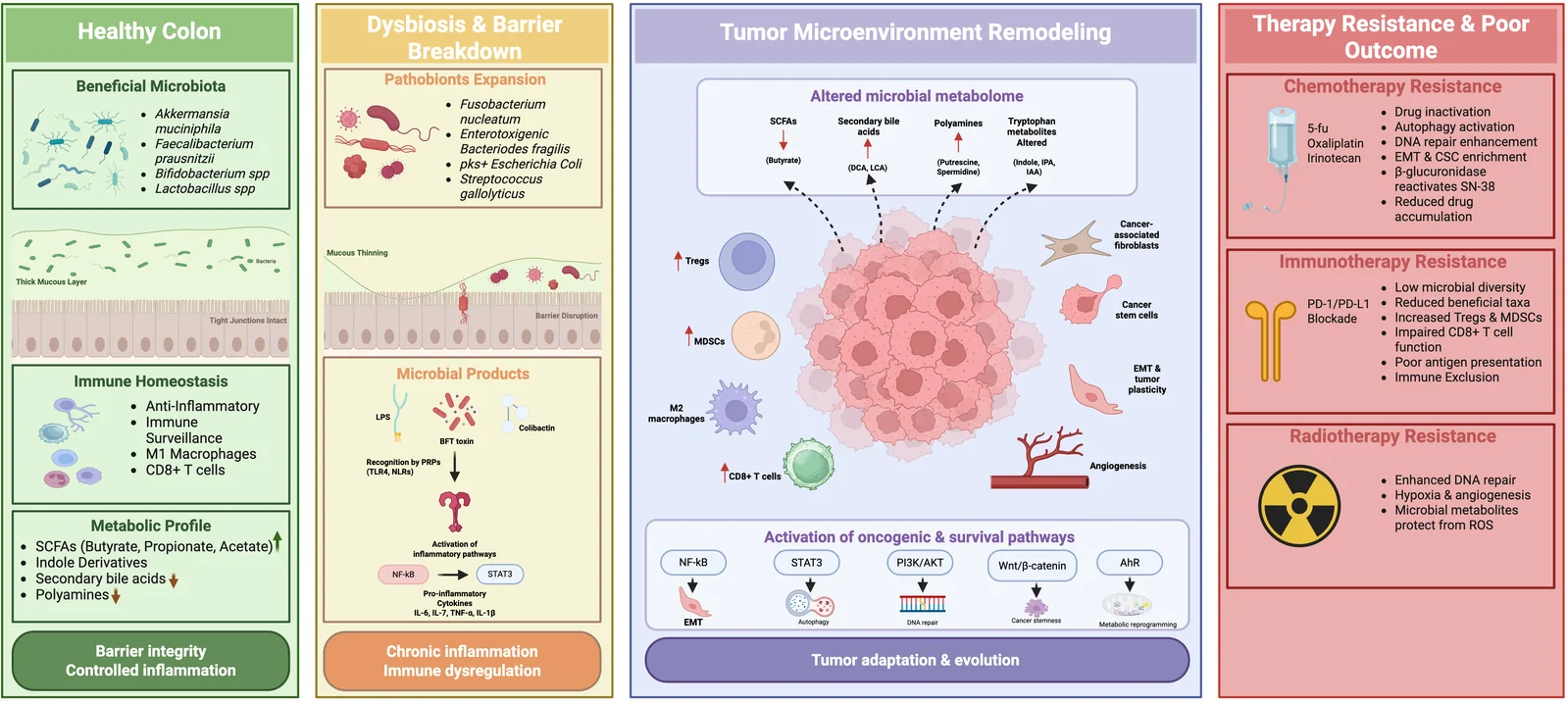
Microbiome-directed therapeutic strategies for overcoming therapy resistance in colorectal cancer. Probiotic supplementation, fecal microbiota transplantation, and dietary or prebiotic interventions restore gut eubiosis by reshaping microbial composition and function. These approaches enhance antitumor immunity, improve responses to chemotherapy and immune checkpoint inhibitors, restore CD8^+^ T-cell activity, and reduce treatment-associated gastrointestinal toxicity.

#### 3.3.1 Targeting microbial xenometabolism and drug degradation

The microbiome acts as a highly diverse, shadow metabolic organ that can chemically modify antineoplastic agents before they exert their therapeutic effect. This is known as xenometabolism, which offers the most immediate and readily translatable therapeutic opportunities.

The best-known example of xenometabolism meeting cancer treatment is the topoisomerase I inhibitor irinotecan. In the liver, the active metabolite SN-38 is detoxified via glucuronidation into the inactive form SN-38G and then excreted into the gastrointestinal tract; however, certain commensal and pathogenic bacteria, such as *E. coli*, *Bacteroides*, and *Clostridium* species, express beta-glucuronidase (113). This enzyme cleaves the glucuronidation moiety, converting SN-38G back to the highly toxic SN-38 in the intestinal lumen. This local reactivation causes very severe dose-limiting diarrhea, which often necessitates premature dose reductions or even stopping treatment altogether. Structural biology has enabled the development of interspecies-selective inhibitors that target specific “bacterial loops” in the beta-glucuronidase active site (114). These targeted inhibitors non-lethally block bacterial enzyme activity without affecting human orthologs or disrupting broader microbiome ecology, and significantly reduce irinotecan-associated enteric toxicity, thereby allowing sustained optimal dosing regimens (115).

The main limitation is that although this mechanism is well established experimentally, microbiome-directed β-glucuronidase inhibition has not yet become a standard component of CRC chemotherapy. Clinical studies must establish whether reducing enteric toxicity translates into improved dose intensity, treatment completion, or survival without producing unintended effects on microbial ecology.

The intratumoral microbiome itself actively degrades therapeutics beyond just the gut lumen. Cytidine deaminase (CDD), an enzyme that rapidly converts pyrimidine nucleoside analogs such as gemcitabine into inactive metabolites, is present at high levels in tumors colonized by Gammaproteobacteria (54). Co-culture of CDD-expressing bacteria with CRC cells directly contributes to chemoresistance, a phenomenon that can be reversed by targeted ciprofloxacin administration (54). Small-molecule CDD inhibitors, including tetrahydrouridine and 3-deazauridine, could synergistically enhance the activity of gemcitabine by altering the competitive nucleotide pools and sensitizing cells that would otherwise be resistant due to high CDD levels (116, 117).

Specifically, some intratumoral *E. coli* isolates from CRC patients have the ability to degrade 5-fluorouracil (5-FU), thereby reducing therapeutic efficacy (118). The co-administration of microbial CDD inhibitors with pyrimidine-based regimens represents a significant therapeutic opportunity to eliminate these intratumoral chemo-sanctuaries (112).

At present, however, these approaches remain largely preclinical, and their clinical development will require demonstration that selective microbial targeting can improve antitumor efficacy without compromising beneficial microbial functions or producing new toxicities.

#### 3.3.2 Dismantling pathogen-induced chemoresistance

Some groups of bacteria, known as pathobionts, have been found to be significantly increased in CRC and contribute to chemoresistance through active interactions with tumor cells. Their removal and disruption of associated signaling pathways are core goals of targeted therapies (119).

For instance, *F. nucleatum* is highly prevalent in CRC and causes significant oxaliplatin and 5-fluorouracil chemoresistance by triggering protective autophagy and suppressing apoptosis. Specifically, *F. nucleatum* triggers the TLR4-MyD88 signaling pathway, resulting in suppression of miR-18a*4802 and the release of transcription factors for the ULK1 and ATG7 genes, which encode proteins required for the initiation of autophagy. Moreover, activation of TLR4 by *F. nucleatum* leads to the NF-κB signal cascade and a subsequent increase in BIRC3 protein levels, an inhibitor of caspases responsible for the apoptotic death of tumor cells. Against oxaliplatin, *F. nucleatum* can induce resistance by inhibiting ferroptosis through the E-cadherin-β-catenin-GPX4 pathway (11, 22). Accordingly, therapeutic approaches targeting TLR4 signaling, autophagy, or ferroptosis may restore chemotherapy sensitivity in CRC.

Other bacteria implicated in resistance in preclinical studies include *pks^+^ E. coli*, which have been associated with EMT-related changes in cancer cells (120). Inhibition of ClbP is considered to impede maturation of precolibactin into genotoxic colibactin. Therefore, ClbP-specific inhibitors could potentially prevent the onset of CRC (121). Nevertheless, these strategies remain predominantly preclinical. Selective depletion of a microbial species may have different effects depending on the surrounding microbial community, and antibiotic or phage-based approaches may alter ecological networks in ways that are not captured in experimental models. Prospective clinical studies are therefore needed before these interventions can be considered therapeutic options in CRC.

The principal microbial species implicated in CRC chemoresistance, their therapeutic agents, and underlying resistance mechanisms are summarized in **Table 1**.

**Table 1:** Microbial mediators of chemoresistance in CRC.

Microbial species	Therapeutic agent	Primary mechanism	Key enzyme/signaling	Evidence level	Reference(s)
*GUS+ E. coli*	Irinotecan	Reactivation of inactive SN-38G to toxic SN-38	beta-glucuronidase (GUS)	Animal models	(115)
Gammaproteobacteria	Gemcitabine	Rapid catabolism into inactive dFdU	Cytidine deaminase (CDD)	Cell-line co-culture and mouse xenografts	(54)
*preTA+ E. coli*	5-Fluorouracil	Local degradation into DHFU	PreA/PreB (preTA operon)	*In vitro*	(118)
*F. nucleatum*	Oxaliplatin	Induction of protective autophagy	TLR4-MyD88 / miR-18a*	Cell lines and xenografts	(11)
*pks+ E. coli*	Various	Induction of EMT and genomic instability	Colibactin genotoxin	Organoid and animal studies	(120)

#### 3.3.3 Microbial metabolites as potential therapeutic agents

In contrast to live microbial therapies, the administration of defined microbial metabolites or microbe-derived molecules may offer a more targeted way to investigate microbiome-associated effects on the TME (122, 123). The metabolites and microbial products evaluated in preclinical CRC studies, their proposed targets, and antitumor mechanisms are summarized in **Table 2**.

**Table 2:** Microbial metabolites and microbe-derived molecules investigated for potential antitumor effects.

Metabolite	Bacterial source	Molecular target	Outcome	Evidence level	Reference(s)
Butyrate	Bacillota	HDACs (Class I/II)	Epigenetic priming; p21 expression	Cell lines and animal models	(124, 125)
Inosine	*A. muciniphila*	A2A Receptor	Th1 differentiation; T-cell rescue	Mouse models; ex vivo T cells	(126, 127)
Ferrichrome	*L. casei*	JNK-CHOP Pathway	ER stress-induced apoptosis	Cell lines and mouse models	(128)
Reuterin	*L. reuteri*	Glutathione (GSH)	Oxidative stress; Translation arrest	Cell lines and mouse models	(129)
Anacardic Acid	*B. caccae*	MAPK / p300 HAT	NET induction; NK cell recruitment	Preclinical	(130)
c-di-AMP	Ruminococcaceae	STING Pathway	Type I IFN; DC maturation	Humanized mouse models	(131)

##### 3.3.3.1 Short-chain fatty acids and epigenetic priming

SCFAs, including butyrate, can influence epithelial and immune-cell function through mechanisms that include histone deacetylase inhibition and signaling through G-protein-coupled receptors such as FFAR2, FFAR3, and GPR109A. Experimental studies have linked these pathways to changes in tumor-suppressor gene expression, epithelial homeostasis, and immune-cell activity. Notably, butyrate has been found to have a dual effect: it can inhibit the growth of some CRC cells while serving as a metabolic substrate for certain effector and memory T-cell populations (124, 132, 133).

These findings provide a mechanistic rationale for further investigating SCFAs as potential therapeutic agents; however, SCFA supplementation has not yet been established as a standard anticancer treatment. Their effects may depend on concentration, cellular metabolic state, and tumor context, emphasizing the need for dose-finding studies and clinical validation.

##### 3.3.3.2 Novel microbial metabolites in preclinical CRC studies

Several microbial metabolites have shown biological activity in experimental CRC systems. For instance, *Lacticaseibacillus casei* (formerly *Lactobacillus casei*), secretes a specific metal chelator, ferrichrome, which induces an acute endoplasmic reticulum (ER) stress response and subsequent selective apoptosis by activating JNK-CHOP pathways in CRC cells (128). *Limosilactobacillus reuteri* (formerly *Lactobacillus reuteri)* synthesizes a highly electrophilic compound, reuterin, which induces selective oxidative stress by reducing cellular glutathione levels and thereby selectively oxidizing ribosomal proteins and halting translation in tumor cells (129). Additionally, *Streptococcus thermophilus* secretes β-galactosidase, which promotes galactose production and alters energy metabolism in CRC cells, increasing oxidative phosphorylation and contributing to reduced tumor-cell proliferation (134). However, most of these findings are derived from cell or animal models. Their clinical translation will require pharmacokinetic characterization, demonstration of tumor-relevant exposure, and confirmation that therapeutic concentrations can be achieved safely in humans.

##### 3.3.3.3 Anacardic acid and innate immunity

Metabolomics profiling of patients who respond positively to immune checkpoint inhibitors (ICIs) shows higher levels of anacardic acid, primarily produced by *Bacteroides caccae*. Anacardic acid activates the classical macrophage activation pathway via MAPK phosphorylation and induces NET formation, promoting the activity of NK cells and cytotoxic T-lymphocytes (130, 135). These observations support an immunomodulatory role for microbial metabolites, but even when a metabolite is more abundant in responders, this association does not establish that the compound is produced by a particular organism *in vivo* or that administering it will improve treatment outcomes. CRC-specific clinical evidence supporting anacardic acid as a predictive biomarker or therapeutic agent remains to be established. Further work should confirm its source, biological activity, exposure, and safety in relevant models and clinical cohorts.

#### 3.3.4 Microbiome and immune checkpoint inhibitor response in CRC

As noted in Section 3.2.3, most CRC cases are pMMR/MSS and derive little benefit from checkpoint inhibitors (136, 137); the immune-exclusion mechanisms involved are described in Section 3.1.5. The microbiome-metabolome axis is increasingly recognized as an important regulator of the tumor immune microenvironment and may contribute to converting these resistant tumors into more immunologically responsive phenotypes, potentially enhancing the activity of PD-1/PD-L1 and CTLA-4 blockade. Importantly, microbial effects on treatment response may be context-dependent; for example, *F. nucleatum* has been linked to chemotherapy resistance but may enhance anti-PD-L1 responses in certain experimental models, highlighting the limitations of single-taxon targeting (138).

##### 3.3.4.1 Metabolic rescue by means of inosine

Inosine, which is synthesized by species such as *A. muciniphila* and *Bifidobacterium pseudolongum*, performs an important function in terms of improving the effects of ICIs (126). It binds to the adenosine A2A receptor (A2AR) expressed by T lymphocytes and induces a signaling cascade via inosine-A2AR-cAMP-PKA, resulting in pCREB. The above effect causes a significant increase in the expression of IL-12Rβ2 and IFN-γ, leading to potent activation of the Th1 phenotype and its accumulation in the tumor microenvironment (127). Additionally, inosine serves as a carbon alternative in the hypoxic, glucose-depleted microenvironment of solid CRC tumors. CD8+ T cells degrade inosine via purine nucleoside phosphorylase (PNP) into hypoxanthine and phosphorylated ribose, thereby generating ATP and precursors for the PPP when glycolysis is not possible due to oxygen deprivation (139). In addition, inosine sensitizes tumor cells to the cytocidal action of T cells by binding and inhibiting the ubiquitin-activating enzyme UBA6 (139). The direction and magnitude of these effects may depend on the cellular context, receptor expression, nutrient availability, and concentration. Although these findings provide a rationale for further study, inosine-based approaches remain experimental; the appropriate dose, delivery strategy, systemic safety, and efficacy in CRC patients have not been established.

##### 3.3.4.2 Antigenic molecular mimicry

The gut microbiota influences CD8+ T cell-based immunity against CRC through molecular mimicry, whereby peptides derived from microorganisms share similarity with antigens expressed on tumor cells. For example, the tape measure protein (TMP1) epitope of a-type 4 (PSMB4) antigen is expressed on tumor cells (140). As a result, CD8+ T cells specific for the viral TMP1 epitope will be able to target PSMB4-expressing tumor cells, significantly boosting the effectiveness of anti-PD-1 therapy. Likewise, the SVYRYYGL (SVY) epitope generated by the bacterium *Bifidobacterium breve* is similar to the SIYRYYGL (SIY) antigen, allowing SVY-specific T cells to target SIY-expressing CRC cells (141). As these approaches depend on antigen-specific immune recognition, their applicability is likely to vary between patients according to HLA type, tumor antigen expression, and baseline microbiome composition. Clinical validation is therefore required before microbiome-derived molecular mimicry can be translated into personalized vaccination or microbial therapies.

##### 3.3.4.3 Fecal microbiota transplantation and defined consortia

FMT represents one of the most direct approaches to therapeutic microbiome remodeling because it can simultaneously alter multiple microbial taxa and functional pathways rather than targeting a single organism or metabolite. Clinical evidence demonstrates that administering FMT from ICI-responding patients into ICI-refractory patients can fundamentally reprogram the systemic immune tone. In early-phase clinical trials, FMT combined with pembrolizumab successfully overcame resistance to anti-PD-1 therapy in previously non-responsive patients (109, 110). Building on these findings, the multicenter Phase II FMT-LUMINate trial demonstrated promising objective response rates following oral capsule FMT administered before immune checkpoint inhibition in both melanoma and non-small cell lung cancer. Interestingly, treatment response was associated with selective depletion of deleterious bacterial taxa rather than donor strain engraftment, suggesting that microbiome remodeling, rather than simple donor colonization, may be the primary mechanism underlying therapeutic benefit (111). These findings are further supported by a recent meta-analysis reporting improved objective response and disease control rates when FMT was combined with immune checkpoint inhibitors, particularly when administered in the first-line setting (142).

However, these studies were not conducted in CRC and therefore provide cross-tumor proof-of-concept rather than evidence of clinical efficacy in CRC. FMT should consequently be considered an investigational microbiome-directed therapy in CRC, with potential applications in immunotherapy sensitization and selected microbiome-mediated toxicities. Although CRC-specific evidence remains limited, these findings support further evaluation of FMT in CRC clinical trials (**Table 3**).

**Table 3:** FMT in cancer therapy and future implications for CRC.

Cancer type	FMT strategy	Major finding	Implications for CRC	Reference(s)
Melanoma	Responder-derived FMT combined with anti-PD-1 (refractory disease); oral capsule FMT before first-line dual ICI	Restored immunotherapy sensitivity in a subset of PD-1-refractory patients with immune activation and microbiome remodeling. First-line capsule FMT also produced high response rates while demonstrating that depletion of detrimental taxa, rather than donor strain engraftment, correlated with benefit.	Provides proof-of-concept that FMT may alter ICI response in selected patients with melanoma. Whether this approach is effective and safe in CRC, particularly MSS/pMMR disease, requires CRC-specific trials.	(109, 110)
Non-small cell lung cancer	Oral capsule FMT before immunotherapy	Objective response rate of approximately 80% with favorable safety. Clinical benefit was associated with microbiome remodeling and depletion of unfavorable bacterial species.	Provides indirect evidence supporting FMT as an immune-priming strategy that could be evaluated alongside checkpoint inhibitors in MSS CRC, but findings cannot establish efficacy in CRC yet.	(111)
Metastatic renal cell carcinoma	Healthy donor FMT plus ICI (Phase I PERFORM); responder-derived donor FMT plus pembrolizumab/axitinib (Phase II TACITO)	FMT was safe, promoted donor microbiome engraftment, increased microbial diversity, prolonged progression-free survival, and improved objective responses in selected patients	Provides indirect information on the feasibility of studying FMT with systemic therapy. The efficacy and safety of this strategy in CRC remain uncertain.	(143, 144)
Gastroesophageal cancer	Allogenic FMT before first-line chemotherapy	Although cachexia was unchanged, FMT improved disease control, prolonged progression-free and overall survival trends, and achieved successful donor engraftment.	Offers a rationale for further study of microbiome modulation alongside systemic therapy, but does not establish benefit in CRC.	(145)

The main limitations of FMT are donor variability, inconsistent engraftment, difficulty defining an optimal microbial composition, potential pathogen transmission, and the absence of standardized preparation and administration protocols. These limitations are particularly important in immunocompromised oncology populations. The FDA has documented serious infections, including a fatal case, following transmission of ESBL-producing *E. coli* through investigational FMT, underscoring the need for rigorous donor screening and microbiological safety procedures (146).

The development of Defined Microbial Consortia (DMCs) provides a more standardized and potentially safer alternative to conventional FMT. VE800, an investigational defined bacterial consortium, has been evaluated clinically in combination with nivolumab in early-phase studies, including cohorts of patients with MSS CRC (147). In a separate phase I trial of the live bacterial product CBM588 in metastatic renal cell carcinoma, the CBM588 arm showed a numerically higher ORR (58% vs. 20%; p=0.06) and significantly longer median PFS (12.7 vs. 2.5 months; p=0.001) (148). These formulations are designed to deliver defined bacterial strains with controlled composition, potentially reducing the unpredictability associated with whole-stool transplantation. However, these approaches remain investigational, and their efficacy, optimal composition, durability of colonization, and long-term safety require further clinical evaluation.

##### 3.3.4.4 Dietary and prebiotic microbiome modulation

Dietary intervention represents a more accessible, non-invasive approach to microbiome modulation. Unlike FMT or engineered microbial therapies, dietary strategies can influence microbial composition and metabolite production without introducing exogenous organisms. High-fiber diets enriched with resistant starch and other fermentable carbohydrates can increase the availability of substrates for SCFA-producing bacteria and thereby alter microbial metabolism. In patients receiving immunotherapy, higher dietary fiber intake has been associated with improved outcomes in melanoma, although these findings cannot be directly extrapolated to CRC (149). The administration of certain soluble fibers, such as inulin and pectin, greatly enhances the effects of anti-PD-1 drugs (131). Pectin treatment has been shown to enhance the effects of anti-PD-1 therapy in humanized CRC mouse models by modulating the gut microbiome (150). Specifically, inulin and pectin enrich the Ruminococcaceae family and *A. muciniphila*, increasing their ability to produce cyclic diadenosine monophosphate (c-di-AMP). This compound serves as a metabolite and a strong activator of the STING pathway, inducing the production of Type I interferon (IFN) in monocytes in the tumors, which stimulates dendritic cell (DC) maturation and activation as well as CD8+ T cell infiltration into the tumor tissues. Inulin-based hydrogel formulations that prolong colonic residence time have shown strong synergistic effects when combined with anti-PD-1 therapy in MSS CRC mouse models (151).

Dietary interventions present significant translational challenges, as microbiome responses to identical dietary substrates vary substantially among individuals. Nutritional status, baseline microbiome composition, treatment-related gastrointestinal toxicity, and the feasibility of maintaining a prescribed diet during chemotherapy can all influence outcomes. Moreover, prospective CRC-specific clinical evidence remains limited. Consequently, dietary manipulation should currently be considered a supportive or investigational strategy rather than a validated method for overcoming treatment resistance.

Collectively, these approaches target different mechanisms that may influence antitumor immunity and are being investigated as potential strategies for treatment-resistant CRC (**Table 4**). Much of the evidence remains preclinical, and the clinical efficacy, safety, and durability of benefit in MSS/pMMR CRC have not been established.

**Table 4:** Overcoming ICI resistance in "Cold" (MSS/pMMR) CRC.

Modality	Key example	Target/antigen	Evidence/clinical trial
Molecular Mimicry	*Enterococcus Phage*	PSMB4 (Tumor Antigen)	(140)
Molecular Mimicry	*B. breve* (SVY)	SIY (Tumor Antigen)	(141)
Defined Consortia	VE800 (11 strains)	CD8+ T cell induction	(147)
Live Supplement	CBM588	SCFA production	(152, 153)
FMT	Responder Stool	Systemic immune tone	(109, 154, 155)

#### 3.3.5 Emerging modalities in high-precision metabolic engineering

Future treatments extend beyond modulation of the native microbiota to include synthetic biology and precision virology approaches capable of selectively targeting and reprogramming the TME.

##### 3.3.5.1 Targeted phage therapy

Unlike broad-spectrum antibiotics, which deplete commensal beneficial pathogens, bacteriophages offer highly selective targeting of resistance-inducing pathobionts. An M5 phage clone was identified by researchers using an M13 phage display library to specifically target *F. nucleatum*. When formulated as silver nanoparticles coated with this M5 phage, the intervention selectively eradicated *F. nucleatum* biofilms in the gut and completely reversed the bacteria’s chemoresistance properties while potentiating the efficacy of folinic acid-5-FU-irinotecan regimens in CRC models (156). The major limitation is that these findings remain preclinical. Phage specificity, bacterial resistance to phages, pharmacokinetics, delivery to the tumor or intestinal niche, and the potential effects of phage-associated formulations on the broader microbiome require evaluation in humans.

##### 3.3.5.2 Synthetic biology and “living” medicines

Advances in genetic engineering have enabled the creation of probiotic strains programmed to perform localized, logic-gated drug delivery directly within the tumor. The synthetic biology strain SYNB1891, a modified *E. coli*, has been engineered to express the STING agonist cyclic adenosine diphosphate ribose (cADPR). Upon colonization of the hypoxic tumor core, the tumor releases cADPR and induces massive localized expression of IFNs without systemic toxicity (157).

##### 3.3.5.3 Metabolic engineering for T-cell survival

L-arginine is strictly required for T-cell survival and antitumor activity but is rapidly depleted in the TME; therefore, engineered probiotic *E. coli* Nissle 1917 (EcN) has been programmed to colonize tumor sites and continuously convert local metabolic ammonia directly into L-arginine. Intratumoral injection of this engineered strain massively increases intracellular L-arginine concentrations, thereby triggering deep intratumoral CD4 and CD8 T cell infiltration, which exerts powerful synergistic antitumor effects when combined with anti-PD-L1 therapies (158).

Other advanced strains of *E. coli* Nissle 1917 have been triple-engineered to directly secrete single-domain antibody fragments against PD-L1 and CTLA-4, accompanied by granulocyte-macrophage colony-stimulating factor for sustained localized checkpoint blockade at the neoplastic site.

Despite their precision, engineered microbial therapies present distinct biosafety challenges, including unintended colonization, horizontal gene transfer, genetic circuit instability, immune clearance, and difficulties controlling bacterial persistence. These challenges are compounded by evolving and non-harmonized regulatory frameworks for living genetic therapeutics, which can complicate the assessment of their safety, manufacturing, and long-term behaviour. Consequently, most engineered microbial therapies remain at the experimental or early clinical development stage, and further regulatory standardization will be essential to facilitate their safe translation into clinical practice (159). Emerging engineered microbial and phage-based therapeutic platforms currently under investigation are summarized in **Table 5**.

**Table 5:** High-precision "living medicines" and engineered modalities.

Engineered chassis	Payload	Delivery trigger	Clinical/research goal	Reference(s)
SYNB1891	STING Agonist	Intratumoral	Localized IFN-I stimulation	(157)
Engineered EcN	L-Arginine	Ammonia conversion	Rescue of T-cell exhaustion	(158)
SLC-EcN	PD-L1/CTLA-4 Abs	Synchronized Lysis	Localized checkpoint blockade	(160)
M5 Phage-Nano	Silver NP	*F. nucleatum* binding	Surgical biofilm eradication	(156)
*Listeria* Vaccine	CD8 T cell primers	Oral delivery	Gut-localized T-cell "stations"	(161)
Iron Nanozyme	Lincomycin	*P. anaerobius*	Reversal of ferroptosis resistance	(162)

### 3.4 Methodological limitations and priorities for future research

Several methodological challenges limit the interpretation and clinical translation of microbiome-metabolome findings in CRC. First, studies use different protocols for stool, blood, and tumor sampling, sample storage, DNA extraction, sequencing, metabolite measurement, and bioinformatic analysis. Standardized collection and processing procedures, appropriate negative controls, and transparent reporting are needed to improve reproducibility across cohorts.

Second, many studies rely on a single sampling time point. Longitudinal sampling before, during, and after treatment would help establish whether microbial or metabolic changes precede treatment failure, arise as a consequence of therapy, or reflect disease progression. This distinction is particularly important because chemotherapy, immunotherapy, surgery, bowel preparation, and antibiotic exposure can themselves alter microbial composition.

Third, stool profiling predominantly characterizes luminal communities and may not accurately represent mucosa-associated or intratumoral microorganisms. Where feasible, future studies should compare stool with matched mucosal and tumor samples and account for spatial heterogeneity within tumors. Low-biomass tumor samples also require rigorous contamination controls. Similarly, measured metabolite abundance should be interpreted in relation to bioavailability, local concentration, host metabolism, and relevant microbial pathways rather than assumed to represent biological activity.

Fourth, diet, medications, antibiotics, bowel transit, geographic and environmental exposures, host genetics, tumor location, disease stage, and prior treatment may confound associations between the microbiome and therapeutic outcomes. These variables should be prospectively recorded and incorporated into study design and analysis. Larger, diverse, independently validated cohorts are needed to determine whether proposed signatures are reproducible across populations and add predictive value beyond established clinical and molecular factors.

Finally, mechanistic studies should distinguish correlation from causation by combining longitudinal human data with appropriately controlled functional experiments. Cell-line and animal-model findings should be identified as preclinical, and findings from other cancer types should be treated as indirect evidence unless confirmed in CRC. Studies of microbiome-directed interventions should also evaluate clinically meaningful outcomes, adverse events, ecological effects, and the durability of microbial modification. These methodological and safety considerations must be addressed before microbiome-derived biomarkers or interventions can be incorporated into routine CRC care.

### 3.5 Future perspectives

Future research should determine whether microbiome-metabolome profiling can improve CRC treatment selection and whether modifying specific microbial communities or metabolites improves clinical outcomes. Emerging approaches increasingly explore the integration of microbiome modulation with immunotherapeutic platforms and next-generation cellular therapies, although their clinical utility in CRC remains to be established (163, 164). Spatial profiling, single-cell approaches, and integrated multi-omics analyses may help characterize interactions between microorganisms, tumor cells, and immune populations that are not captured by stool profiling alone (12, 97, 165). Findings from these approaches will require validation in appropriate clinical settings before they can guide treatment decisions.

Clinical studies should evaluate microbiome-directed interventions according to their specific mechanisms and levels of supporting evidence. Approaches such as FMT, defined microbial consortia, dietary modulation, microbial metabolites, phage-based therapies, and engineered microorganisms remain investigational for overcoming CRC treatment resistance unless supported by CRC-specific clinical evidence. Ongoing early-phase trials evaluating microbiome-based interventions in combination with immune checkpoint inhibitors may help clarify their feasibility, safety, and preliminary efficacy (166). However, findings should be interpreted according to the cancer type studied and should not be extrapolated directly to CRC without supporting evidence.

Future trials should prespecify clinically meaningful outcomes and assess adverse events alongside evidence of biological activity, such as microbial engraftment where relevant and changes in metabolite exposure. They should also evaluate the durability of microbiome modification and whether observed biological changes are associated with treatment benefit. Candidate biomarkers and prediction models will require prospective evaluation and independent validation against established clinical and molecular predictors.

Progress toward microbiome-informed precision oncology will depend on reproducible evidence linking microbial or metabolic features to clinically meaningful outcomes and demonstrating that candidate interventions provide a favorable balance of benefit and risk. Until such evidence is established, microbiome and metabolome profiling and most microbiome-directed interventions should be considered investigational rather than routine components of CRC treatment (**Figure 4**).

**Figure 4:**
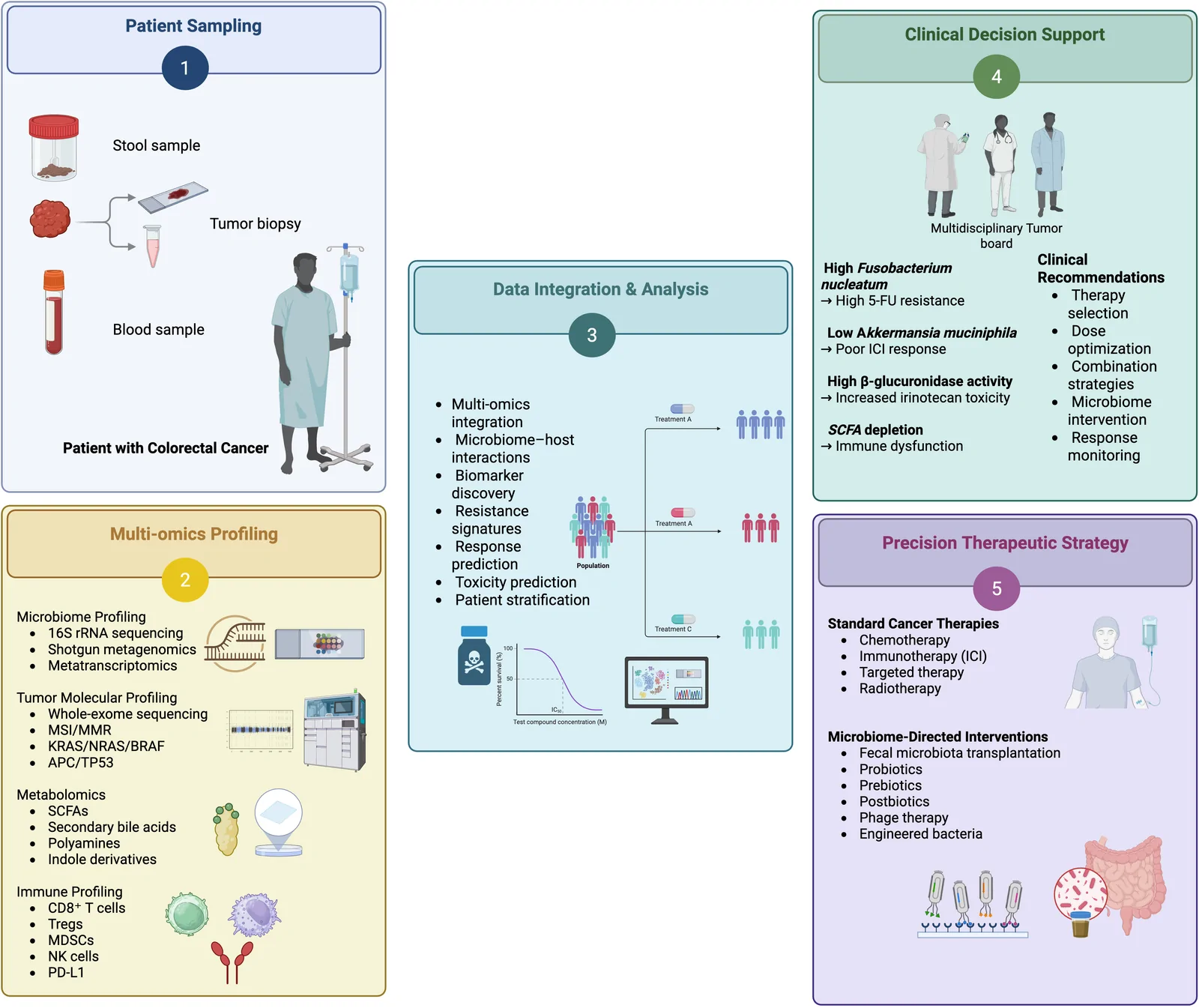
Multi-omics-guided precision therapeutic framework for microbiome-mediated therapy resistance in colorectal cancer. Stool, blood, and tumor samples undergo integrated microbiome, genomic, metabolomic, and immune profiling. Artificial intelligence and multi-omics data integration identify resistance-associated biomarkers, enable patient stratification, and support clinical decision-making. These insights guide personalized combinations of conventional anticancer therapies and microbiome-directed interventions to optimize treatment response and minimize toxicity.

## 4 Conclusion

The microbiome-metabolome axis is increasingly being recognized as a possible key driver of therapy resistance in CRC, influencing treatment response through interconnected inflammatory, immune, metabolic, and pharmacologic mechanisms. Experimental studies have identified possible links between selected microorganisms, microbial metabolites, and treatment response; however, the strength of evidence varies across mechanisms, and associations observed in human cohorts do not necessarily establish causality. Emerging approaches, including microbiome-based biomarkers, dietary modulation, FMT, defined microbial consortia, microbial metabolites, and engineered microbial therapies, offer potential avenues for further investigation. Future research should prioritize longitudinal studies, prospective validation, and carefully designed clinical trials to determine whether microbiome–metabolome profiling can improve treatment selection and whether targeted modification of these systems can meaningfully improve patient outcomes. Until such evidence is established, microbiome-informed approaches should be considered promising research directions rather than established strategies for overcoming CRC therapy resistance.

## References

[1] Sung H Ferlay J Siegel RL Laversanne M Soerjomataram I Jemal A . Global cancer statistics 2020: GLOBOCAN estimates of incidence and mortality worldwide for 36 cancers in 185 countries. CA Cancer J Clin. (2021) 71:209–49. doi: 10.3322/caac.21660 33538338

[2] National Cancer Institute . Cancer Stat Facts: Colorectal Cancer (2025). Available online at: https://seer.cancer.gov/statfacts/html/colorect.html (Accessed June 14, 2026).

[3] Siegel RL Miller KD Goding Sauer A Fedewa SA Butterly LF Anderson JC . Colorectal cancer statistics, 2020. CA Cancer J Clin. (2020) 70:145–64. doi: 10.3322/caac.21601 32133645

[4] Venugopal A Carethers JM . Epidemiology and biology of early onset colorectal cancer. Excli J. (2022) 21:162–82. doi: 10.17179/EXCLI2021-4456 35221839 PMC8859644

[5] Wong SH Yu J . Gut microbiota in colorectal cancer: mechanisms of action and clinical applications. Nat Rev Gastroenterol Hepatol. (2019) 16:690–704. doi: 10.1038/s41575-019-0209-8 31554963

[6] Louis P Hold GL Flint HJ . The gut microbiota, bacterial metabolites and colorectal cancer. Nat Rev Microbiol. (2014) 12:661–72. doi: 10.1038/nrmicro3344 25198138

[7] Akimoto N Ugai T Zhong R Hamada T Fujiyoshi K Giannakis M . Rising incidence of early-onset colorectal cancer — a call to action. Nat Rev Clin Oncol. (2021) 18:230–43. doi: 10.1038/s41571-020-00445-1 33219329 PMC7994182

[8] Zhang H Tian Y Xu C Chen M Xiang Z Gu L . Crosstalk between gut microbiotas and fatty acid metabolism in colorectal cancer. Cell Death Discov. (2025) 11:78. doi: 10.1038/s41420-025-02364-5 40011436 PMC11865559

[9] Schwabe RF Jobin C . The microbiome and cancer. Nat Rev Cancer. (2013) 13:800–12. doi: 10.1038/nrc3610 24132111 PMC3986062

[10] Sepich-Poore GD Zitvogel L Straussman R Hasty J Wargo JA Knight R . The microbiome and human cancer. Science. (2021) 371:eabc4552. doi: 10.1126/science.abc4552 33766858 PMC8767999

[11] Yu T Guo F Yu Y Sun T Ma D Han J . Fusobacterium nucleatum promotes chemoresistance to colorectal cancer by modulating autophagy. Cell. (2017) 170:548–563.e16. doi: 10.1016/j.cell.2017.07.008 28753429 PMC5767127

[12] Tudorache M Treteanu A-R Gradisteanu Pircalabioru G Lixandru-Petre I-O Bolocan A Andronic O . Gut microbiome alterations in colorectal cancer: mechanisms, therapeutic strategies, and precision oncology perspectives. Cancers. (2025) 17:2294. doi: 10.3390/cancers17142294 40723178 PMC12293209

[13] Soomro MY Khan SR Muhammad H Ahmad S Zehra N Ali I . Exploring treatment outcomes in Stage II-III rectal cancer patients undergoing neoadjuvant therapy at a tertiary care center in Pakistan: a comprehensive analysis of pathological outcomes. BMC Cancer. (2024) 24:479. doi: 10.1186/s12885-024-12241-6 38627736 PMC11020334

[14] Shoaib D Soomro MY Khan AF Khan HA Suleman S Moosajee M . Primary anorectal melanoma at a South Asian tertiary care center: a single-institution brief report. SN Compr Clin Med. (2026) 8:94. doi: 10.1007/s42399-026-02358-z30311153

[15] Tito RY Verbandt S Aguirre Vazquez M Lahti L Verspecht C Lloréns-Rico V . Microbiome confounders and quantitative profiling challengepredicted microbial targets in colorectal cancer development. Nat Med. (2024) 30:1339–48. doi: 10.1038/s41591-024-02963-2 38689063 PMC11108775

[16] Sayin SI Wahlström A Felin J Jäntti S Marschall H-U Bamberg K . Gut microbiota regulates bile acid metabolism by reducing the levels of tauro-beta-muricholic acid, a naturally occurring FXR antagonist. Cell Metab. (2013) 17:225–35. doi: 10.1016/j.cmet.2013.01.003 23395169

[17] Min S Zhang Y Zhang H Liu Q . Gut microbiota drives cancer evolution and therapy resistance. Mol Cancer. (2026) 25:39. doi: 10.1186/s12943-025-02564-8 41527087 PMC12918593

[18] Bai B Ma J Xu W Chen X Chen X Lv C . Gut microbiota and colorectal cancer: mechanistic insights, diagnostic advances, and microbiome-based therapeutic strategies. Front Microbiol. (2025) 16:1699893. doi: 10.3389/fmicb.2025.1699893 41356485 PMC12675188

[19] Koh A De Vadder F Kovatcheva-Datchary P Bäckhed F . From dietary fiber to host physiology: short-chain fatty acids as key bacterial metabolites. Cell. (2016) 165:1332–45. doi: 10.1016/j.cell.2016.05.041 27259147

[20] Venkatesh M Mukherjee S Wang H Li H Sun K Benechet AP . Symbiotic bacterial metabolites regulate gastrointestinal barrier function via the xenobiotic sensor PXR and Toll-like receptor 4. Immunity. (2014) 41:296–310. doi: 10.1016/j.immuni.2014.06.014 25065623 PMC4142105

[21] Sears CL Garrett WS . Microbes, microbiota, and colon cancer. Cell Host Microbe. (2014) 15:317–28. doi: 10.1016/j.chom.2014.02.007 24629338 PMC4003880

[22] Rubinstein MR Wang X Liu W Hao Y Cai G Han YW . Fusobacterium nucleatum promotes colorectal carcinogenesis by modulating E-cadherin/β-catenin signaling via its FadA adhesin. Cell Host Microbe. (2013) 14:195–206. doi: 10.1016/j.chom.2013.07.012 23954158 PMC3770529

[23] Boleij A Hechenbleikner EM Goodwin AC Badani R Stein EM Lazarev MG . The Bacteroides fragilis toxin gene is prevalent in the colon mucosa of colorectal cancer patients. Clin Infect Dis. (2015) 60:208–15. doi: 10.1093/cid/ciu787 25305284 PMC4351371

[24] Gu M Yin W Zhang J Yin J Tang X Ling J . Role of gut microbiota and bacterial metabolites in mucins of colorectal cancer. Front Cell Infect Microbiol. (2023) 13:1119992. doi: 10.3389/fcimb.2023.1119992 37265504 PMC10229905

[25] Rebersek M . Gut microbiome and its role in colorectal cancer. BMC Cancer. (2021) 21:1325. doi: 10.1186/s12885-021-09054-2 34895176 PMC8666072

[26] Huang M Zhang Y Chen Z Yu X Luo S Peng X . Gut microbiota reshapes the TNBC immune microenvironment: emerging immunotherapeutic strategies. Pharmacol Res. (2025) 215:107726. doi: 10.1016/j.phrs.2025.107726 40184763

[27] Spanogiannopoulos P Bess EN Carmody RN Turnbaugh PJ . The microbial pharmacists within us: a metagenomic view of xenobiotic metabolism. Nat Rev Microbiol. (2016) 14:273–87. doi: 10.1038/nrmicro.2016.17 26972811 PMC5243131

[28] Mohseni AH Taghinezhad-S S Casolaro V Lv Z Li D . Potential links between the microbiota and T cell immunity determine the tumor cell fate. Cell Death Dis. (2023) 14:154. doi: 10.1038/s41419-023-05560-2 36828830 PMC9958015

[29] Oh B Boyle F Pavlakis N Clarke S Eade T Hruby G . The gut microbiome and cancer immunotherapy: can we use the gut microbiome as a predictive biomarker for clinical response in cancer immunotherapy? Cancers. (2021) 13:4824. doi: 10.3390/cancers13194824 34638308 PMC8508052

[30] Sillo TO Beggs AD Middleton G Akingboye A . The gut microbiome, microsatellite status and the response to immunotherapy in colorectal cancer. Int J Mol Sci. (2023) 24:5767. doi: 10.3390/ijms24065767 36982838 PMC10054450

[31] Li S Che C Zhou Y Fan D Bai X Lu Y . Gut microecology empowers cancer immunotherapy: commensal microbiota-mediated mechanisms and translational prospects of PD-1/PD-L1 therapy. Cancer Biol Med. (2026) 23:60–77. doi: 10.20892/j.issn.2095-3941.2025.0347 41612728 PMC12911435

[32] Chen DS Mellman I . Elements of cancer immunity and the cancer–immune set point. Nature. (2017) 541:321–30. doi: 10.1038/nature21349 28102259

[33] Kang Y-B Cai Y . Faecal microbiota transplantation enhances efficacy of immune checkpoint inhibitors therapy against cancer. World J Gastroenterol. (2021) 27:5362–75. doi: 10.3748/wjg.v27.i32.5362 34539138 PMC8409158

[34] Longley DB Harkin DP Johnston PG . 5-Fluorouracil: mechanisms of action and clinical strategies. Nat Rev Cancer. (2003) 3:330–8. doi: 10.1038/nrc1074 12724731

[35] Grivicich I Mans DRA Peters GJ Schwartsmann G . Irinotecan and oxaliplatin: an overview of the novel chemotherapeutic options for the treatment of advanced colorectal cancer. Braz J Med Biol Res. (2001) 34:1087–103. doi: 10.1590/S0100-879X2001000900001 11514832

[36] Dasari S Bernard Tchounwou P . Cisplatin in cancer therapy: molecular mechanisms of action. Eur J Pharmacol. (2014) 740:364–78. doi: 10.1016/j.ejphar.2014.07.025 25058905 PMC4146684

[37] Eiman L Moazzam K Anjum S Kausar H Sharif EAM Ibrahim WN . Gut dysbiosis in cancer immunotherapy: microbiota-mediated resistance and emerging treatments. Front Immunol. (2025) 16:1575452. doi: 10.3389/fimmu.2025.1575452 40927726 PMC12415008

[38] Li Y Shen X Wang D Sun K . The gut microbiome in colorectal cancer: mechanisms of carcinogenesis and emerging microbiota-targeted therapies. Discov Oncol. (2026) 17:181. doi: 10.1007/s12672-025-04367-1 41498910 PMC12858699

[39] Meng C Bai C Brown TD Hood LE Tian Q . Human gut microbiota and gastrointestinal cancer. Genomics Proteomics Bioinf. (2018) 16:33–49. doi: 10.1016/j.gpb.2017.06.002 29474889 PMC6000254

[40] Ternes D Karta J Tsenkova M Wilmes P Haan S Letellier E . Microbiome in colorectal cancer: how to get from meta-omics to mechanism? Trends Microbiol. (2020) 28:401–23. doi: 10.1016/j.tim.2020.01.001 32298617

[41] Grivennikov SI Karin M . Dangerous liaisons: STAT3 and NF-κB collaboration and crosstalk in cancer. Cytokine Growth Factor Rev. (2010) 21:11–9. doi: 10.1016/j.cytogfr.2009.11.005 20018552 PMC2834864

[42] Li F Sethi G . Targeting transcription factor NF-κB to overcome chemoresistance and radioresistance in cancer therapy. Biochim Biophys Acta BBA - Rev Cancer. (2010) 1805:167–80. doi: 10.1016/j.bbcan.2010.01.002 20079806

[43] Qu Y Dou B Tan H Feng Y Wang N Wang D . Tumor microenvironment-driven non-cell-autonomous resistance to antineoplastic treatment. Mol Cancer. (2019) 18:69. doi: 10.1186/s12943-019-0992-4 30927928 PMC6441162

[44] Liu T Zhang L Joo D Sun S-C . NF-κB signaling in inflammation. Signal Transduct Target Ther. (2017) 2:17023. doi: 10.1038/sigtrans.2017.23 29158945 PMC5661633

[45] West NR McCuaig S Franchini F Powrie F . Emerging cytokine networks in colorectal cancer. Nat Rev Immunol. (2015) 15:615–29. doi: 10.1038/nri3896 26358393

[46] Bardelčíková A Šoltys J Mojžiš J . Oxidative stress, inflammation and colorectal cancer: an overview. Antioxidants. (2023) 12:901. doi: 10.3390/antiox12040901 37107276 PMC10135609

[47] Hanahan D Weinberg RA . Hallmarks of cancer: the next generation. Cell. (2011) 144:646–74. doi: 10.1016/j.cell.2011.02.013 21376230

[48] Mehmood MS Danaf N . Bacteroides fragilis toxin in colorectal tumors activates STAT3 and drives microsatellite instability. Ann Med Surg. (2026) 88:957–8. doi: 10.1097/MS9.0000000000004287 41497166 PMC12768026

[49] Yu H Pardoll D Jove R . STATs in cancer inflammation and immunity: a leading role for STAT3. Nat Rev Cancer. (2009) 9:798–809. doi: 10.1038/nrc2734 19851315 PMC4856025

[50] De Simone V Franzè E Ronchetti G Colantoni A Fantini MC Di Fusco D . Th17-type cytokines, IL-6 and TNF-α synergistically activate STAT3 and NF-kB to promote colorectal cancer cell growth. Oncogene. (2015) 34:3493–503. doi: 10.1038/onc.2014.286 25174402 PMC4493653

[51] Wang T Song P Zhong T Wang X Xiang X Liu Q . The inflammatory cytokine IL-6 induces FRA1 deacetylation promoting colorectal cancer stem-like properties. Oncogene. (2019) 38:4932–47. doi: 10.1038/s41388-019-0763-0 30804456 PMC6756002

[52] Grivennikov SI Wang K Mucida D Stewart CA Schnabl B Jauch D . Adenoma-linked barrier defects and microbial products drive IL-23/IL-17-mediated tumour growth. Nature. (2012) 491:254–8. doi: 10.1038/nature11465 23034650 PMC3601659

[53] Florescu DN Boldeanu M-V Verban R-E Florescu LM Serbanescu M-S Ionescu M . Correlation of the pro-inflammatory cytokines IL-1β, IL-6, and TNF-α, inflammatory markers, and tumor markers with the diagnosis and prognosis of colorectal cancer. Life. (2023) 13:2261. doi: 10.3390/life13122261 38137862 PMC10744550

[54] Geller LT Barzily-Rokni M Danino T Jonas OH Shental N Nejman D . Potential role of intratumor bacteria in mediating tumor resistance to the chemotherapeutic drug gemcitabine. Science. (2017) 357:1156–60. doi: 10.1126/science.aah5043 28912244 PMC5727343

[55] Mima K Nishihara R Qian ZR Cao Y Sukawa Y Nowak JA . *Fusobacterium nucleatum* in colorectal carcinoma tissue and patient prognosis. Gut. (2016) 65:1973–80. doi: 10.1136/gutjnl-2015-310101 26311717 PMC4769120

[56] Wang N Zhang L Leng X-X Xie Y-L Kang Z-R Zhao L-C . *Fusobacterium nucleatum* induces chemoresistance in colorectal cancer by inhibiting pyroptosis via the Hippo pathway. Gut Microbes. (2024) 16:2333790. doi: 10.1080/19490976.2024.2333790 38533566 PMC10978024

[57] Zhang X Huang J Shang G Lu Y Liu H Zhang Y . Multiomics profiling reveals that P. gingivalis-induced extracellular vesicle reprogramming promotes immune evasion in colorectal cancer through autophagy-mediated STING degradation. Front Cell Infect Microbiol. (2025) 15:1604554. doi: 10.3389/fcimb.2025.1604554 41170290 PMC12568708

[58] Olsen I Yilmaz Ö . Possible role of *Porphyromonas gingivalis* in orodigestive cancers. J Oral Microbiol. (2019) 11:1563410. doi: 10.1080/20002297.2018.1563410 30671195 PMC6327928

[59] Yuan K Xu S Liu G Han Y Hu J Zhang W . *Porphyromonas gingivalis* promotes oral squamous cell carcinoma progression by modulating autophagy. Oral Dis. (2025) 31:492–502. doi: 10.1111/odi.15157 39435608

[60] Guo C Wang C . Microbiota-host metabolism reprogramming in colorectal cancer: from pathogenesis to precision therapies. Front Oncol. (2025) 15:1713614. doi: 10.3389/fonc.2025.1713614 41415548 PMC12708274

[61] Grion BAR Fonseca PLC Kato RB García GJY Vaz ABM Jiménez BN . Identification of taxonomic changes in the fecal bacteriome associated with colorectal polyps and cancer: potential biomarkers for early diagnosis. Front Microbiol. (2024) 14:1292490. doi: 10.3389/fmicb.2023.1292490 38293554 PMC10827328

[62] Casero RA Murray Stewart T Pegg AE . Polyamine metabolism and cancer: treatments, challenges and opportunities. Nat Rev Cancer. (2018) 18:681–95. doi: 10.1038/s41568-018-0050-3 30181570 PMC6487480

[63] Lyons NJ Giri R Begun J Clark D Proud D He Y . Reactive oxygen species as mediators of disease progression and therapeutic response in colorectal cancer. Antioxid Redox Signal. (2023) 39:186–205. doi: 10.1089/ars.2022.0127 36792932

[64] Zaurito AE Tschurtschenthaler M . Microenvironmental metabolites in the intestine: messengers between health and disease. Metabolites. (2022) 12:46. doi: 10.3390/metabo12010046 35050167 PMC8778376

[65] Liu Y Lau H-H Yu J . Microbial metabolites in colorectal tumorigenesis and cancer therapy. Gut Microbes. (2023) 15:2203968. doi: 10.1080/19490976.2023.2203968 37095682 PMC10132243

[66] Xie H Xu H Wu J Yang J Zeng W Jin S . Effect of indole 3-propionic acid from microbiota on the immunity of CD8^+^ T cell to improve chemotherapy of colorectal cancer. J Clin Oncol. (2024) 42:e15194–4. doi: 10.1200/JCO.2024.42.16_suppl.e1519442852411

[67] Li Y Wu Q Zhang J Xun Q . Gut microbial metabolites in inflammation-associated colorectal cancer: mechanisms and therapeutic implications. Front Microbiol. (2026) 17:1802686. doi: 10.3389/fmicb.2026.1802686 42146051 PMC13176243

[68] Mayer IA Arteaga CL . The PI3K/AKT pathway as a target for cancer treatment. Annu Rev Med. (2016) 67:11–28. doi: 10.1146/annurev-med-062913-051343 26473415

[69] Feng L Wang M Li X Wu L Gu D Zhang B . Integrative subtyping by bile acid metabolism identifies CLCA1/UGT2A3/ZG16 as markers of immune dysfunction and poor prognosis in colorectal cancer. Front Oncol. (2026) 15:1739534. doi: 10.3389/fonc.2025.1739534 41602387 PMC12832251

[70] Corrêa‐Oliveira R Fachi JL Vieira A Sato FT Vinolo MAR . Regulation of immune cell function by short‐chain fatty acids. Clin Transl Immunol. (2016) 5:e73. doi: 10.1038/cti.2016.17 27195116 PMC4855267

[71] Yoo HY Park SY Chang S-Y Kim SH . Regulation of butyrate-induced resistance through AMPK signaling pathway in human colon cancer cells. Biomedicines. (2021) 9:1604. doi: 10.3390/biomedicines9111604 34829834 PMC8615665

[72] He Y Ling Y Zhang Z Mertens RT Cao Q Xu X . Butyrate reverses ferroptosis resistance in colorectal cancer by inducing c-Fos-dependent xCT suppression. Redox Biol. (2023) 65:102822. doi: 10.1016/j.redox.2023.102822 37494767 PMC10388208

[73] Kim NH Song SH Choi YH Hwang KH Yun JS Song H . Competing endogenous RNA of Snail and Zeb1 UTR in therapeutic resistance of colorectal cancer. Int J Mol Sci. (2021) 22:9589. doi: 10.3390/ijms22179589 34502497 PMC8431469

[74] Brabletz T Kalluri R Nieto MA Weinberg RA . EMT in cancer. Nat Rev Cancer. (2018) 18:128–34. doi: 10.1038/nrc.2017.118 29326430

[75] Chen C Su Q Zi M Hua X Zhang Z . Harnessing gut microbiota for colorectal cancer therapy: from clinical insights to therapeutic innovations. NPJ Biofilms Microbiomes. (2025) 11:190. doi: 10.1038/s41522-025-00818-3 41028744 PMC12485087

[76] Chen Z Li Y Zhang C Yi H Wu C Wang J . Downregulation of Beclin 1 and impairment of autophagy in a small population of colorectal cancer. Dig Dis Sci. (2013) 58:2887–94. doi: 10.1007/s10620-013-2732-8 23812859 PMC3781302

[77] Vescovo T Bontempi G Bayat M Piredda L Fidaleo M Strippoli R . Gut microbiota interplay with autophagy-EMT dynamics in colorectal cancer. Front Cell Dev Biol. (2025) 13:1608248. doi: 10.3389/fcell.2025.1608248 40917756 PMC12408621

[78] Zhang W Jin C Liu S Wan X Li Y Liu J . Myeloid-derived suppressor cells and regulatory T cells in colorectal cancer: a synergistic immunosuppressive axis and emerging therapeutic opportunities. Front Immunol. (2026) 17:1757513. doi: 10.3389/fimmu.2026.1757513 41694372 PMC12894373

[79] Gur C Ibrahim Y Isaacson B Yamin R Abed J Gamliel M . Binding of the Fap2 protein of Fusobacterium nucleatum to human inhibitory receptor TIGIT protects tumors from immune cell attack. Immunity. (2015) 42:344–55. doi: 10.1016/j.immuni.2015.01.010 25680274 PMC4361732

[80] Liu Y Wong CC Ding Y Gao M Wen J Lau H-H . Peptostreptococcus anaerobius mediates anti-PD1 therapy resistance and exacerbates colorectal cancer via myeloid-derived suppressor cells in mice. Nat Microbiol. (2024) 9:1467–82. doi: 10.1038/s41564-024-01695-w 38750176 PMC11153135

[81] Jiang S-S Xie Y-L Xiao X-Y Kang Z-R Lin X-L Zhang L . Fusobacterium nucleatum-derived succinic acid induces tumor resistance to immunotherapy in colorectal cancer. Cell Host Microbe. (2023) 31:781–797.e9. doi: 10.1016/j.chom.2023.04.010 37130518

[82] Liao Q Zhou X Wu L Yang Y Zhu X Liao H . Gut microbial metabolite 4-hydroxybenzeneacetic acid drives colorectal cancer progression via accumulation of immunosuppressive PMN-MDSCs. J Clin Invest. (2025) 135:e181243. doi: 10.1172/JCI181243 40179015 PMC12126219

[83] Luke JJ Bao R Sweis RF Spranger S Gajewski TF . WNT/β-catenin pathway activation correlates with immune exclusion across human cancers. Clin Cancer Res. (2019) 25:3074–83. doi: 10.1158/1078-0432.CCR-18-1942 30635339 PMC6522301

[84] Sivan A Corrales L Hubert N Williams JB Aquino-Michaels K Earley ZM . Commensal *Bifidobacterium* promotes antitumor immunity and facilitates anti–PD-L1 efficacy. Science. (2015) 350:1084–9. doi: 10.1126/science.aac4255 26541606 PMC4873287

[85] Routy B Le Chatelier E Derosa L Duong CPM Alou MT Daillère R . Gut microbiome influences efficacy of PD-1–based immunotherapy against epithelial tumors. Science. (2018) 359:91–7. doi: 10.1126/science.aan3706 29097494

[86] Vétizou M Pitt JM Daillère R Lepage P Waldschmitt N Flament C . Anticancer immunotherapy by CTLA-4 blockade relies on the gut microbiota. Science. (2015) 350:1079–84. doi: 10.1126/science.aad1329 26541610 PMC4721659

[87] Mahdy MS Azmy AF Dishisha T Mohamed WR Ahmed KA Hassan A . Irinotecan-gut microbiota interactions and the capability of probiotics to mitigate irinotecan-associated toxicity. BMC Microbiol. (2023) 23:53. doi: 10.1186/s12866-023-02791-3 36864380 PMC9979425

[88] Bhatt AP Pellock SJ Biernat KA Walton WG Wallace BD Creekmore BC . Targeted inhibition of gut bacterial β-glucuronidase activity enhances anticancer drug efficacy. Proc Natl Acad Sci. (2020) 117:7374–81. doi: 10.1073/pnas.1918095117 32170007 PMC7132129

[89] Khan SR Scheffler M Soomar SM Rashid YA Moosajee M Ahmad A . Role of circulating-tumor DNA in the early-stage non-small cell lung carcinoma as a predictive biomarker. Pathol Res Pract. (2023) 245:154455. doi: 10.1016/j.prp.2023.154455 37054576

[90] Holjak E Brasoveanu T Verma S Khan S Black M Dhaliwal I . Circulating tumor DNA as part of the routine work-up for patients with suspected advanced lung cancer. J Liq Biopsy. (2025) 10:100443. doi: 10.1016/j.jlb.2025.100443 41323435 PMC12657812

[91] Takenaka IKTM Bartelli TF Defelicibus A Sendoya JM Golubicki M Robbio J . Exome and tissue-associated microbiota as predictive markers of response to neoadjuvant treatment in locally advanced rectal cancer. Front Oncol. (2022) 12:809441. doi: 10.3389/fonc.2022.809441 35392220 PMC8982181

[92] Chen F Dai X Zhou C-C Li K Zhang Y Lou X-Y . Integrated analysis of the faecal metagenome and serum metabolome reveals the role of gut microbiome-associated metabolites in the detection of colorectal cancer and adenoma. Gut. (2022) 71:1315–25. doi: 10.1136/gutjnl-2020-323476 34462336 PMC9185821

[93] Bisht V Nash K Xu Y Agarwal P Bosch S Gkoutos GV . Integration of the microbiome, metabolome and transcriptomics data identified novel metabolic pathway regulation in colorectal cancer. Int J Mol Sci. (2021) 22:5763. doi: 10.3390/ijms22115763 34071236 PMC8198673

[94] Coker OO Liu C Wu WKK Wong SH Jia W Sung JJY . Altered gut metabolites and microbiota interactions are implicated in colorectal carcinogenesis and can be non-invasive diagnostic biomarkers. Microbiome. (2022) 10:35. doi: 10.1186/s40168-021-01208-5 35189961 PMC8862353

[95] Qiu X Wang A Wang J Zhang Z Tao L . Mitochondrial metabolic reprogramming in colorectal cancer: mechanisms of resistance and future clinical interventions. Cell Death Discov. (2025) 11:375. doi: 10.1038/s41420-025-02670-y 40783392 PMC12335542

[96] Zhao L-Y Mei J-X Yu G Lei L Zhang W-H Liu K . Role of the gut microbiota in anticancer therapy: from molecular mechanisms to clinical applications. Signal Transduct Target Ther. (2023) 8:201. doi: 10.1038/s41392-023-01406-7 37179402 PMC10183032

[97] Zhang C Wang Y Cheng L Cao X Liu C . Gut microbiota in colorectal cancer: a review of its influence on tumor immune surveillance and therapeutic response. Front Oncol. (2025) 15:1557959. doi: 10.3389/fonc.2025.1557959 40110192 PMC11919680

[98] Yu Y Zhao W Yang M Wu B Yuan X . Tumor-promoting gut microbes in colorectal cancer: mechanisms and translational perspectives. Int J Med Sci. (2026) 23:63–75. doi: 10.7150/ijms.123494 41399387 PMC12701968

[99] Jiang S Geng S Chen Q Zhang C Cheng M Yu Y . Effects of concomitant antibiotics use on immune checkpoint inhibitor efficacy in cancer patients. Front Oncol. (2022) 12:823705. doi: 10.3389/fonc.2022.823705 35223505 PMC8864310

[100] Eysha M Fouani M Munir M Black M Long A Elchouemi M . The intricate interplay of microbial metabolomics and carcinogenesis: a spotlight on mechanistic pathways, clinical implications and methodological challenges. Front Cell Infect Microbiol. (2026) 16:1787954. doi: 10.3389/fcimb.2026.1787954 42291307 PMC13259796

[101] Spanogiannopoulos P Kyaw TS Guthrie BGH Bradley PH Lee JV Melamed J . Host and gut bacteria share metabolic pathways for anti-cancer drug metabolism. Nat Microbiol. (2022) 7:1605–20. doi: 10.1038/s41564-022-01226-5 36138165 PMC9530025

[102] Trepka KR Kidder WA Kyaw TS Halsey T Olson CA Ortega EF . Expansion of a bacterial operon during cancer treatment ameliorates fluoropyrimidine toxicity. Sci Transl Med. (2025) 17:eadq8870. doi: 10.1126/scitranslmed.adq8870 40238917 PMC12087798

[103] Ding X Ting N-N Wong CC Huang P Bakuradze N Jiang L . Bacteroides fragilis promotes chemoresistance in colorectal cancer, and its elimination by phage VA7 restores chemosensitivity. Cell Host Microbe. (2025) 33:941–956.e10. doi: 10.1016/j.chom.2025.05.004 40446807

[104] Shuwen H Yangyanqiu W Jian C Boyang H Gong C Jing Z . Synergistic effect of sodium butyrate and oxaliplatin on colorectal cancer. Transl Oncol. (2023) 27:101598. doi: 10.1016/j.tranon.2022.101598 36512976 PMC9763735

[105] Li Y He P Chen Y Hu J Deng B Liu C . Microbial metabolite sodium butyrate enhances the anti-tumor efficacy of 5-fluorouracil against colorectal cancer by modulating PINK1/Parkin signaling and intestinal flora. Sci Rep. (2024) 14:13063. doi: 10.1038/s41598-024-63993-x 38844824 PMC11156851

[106] Ajab SM Zoughbor SH Labania LA Östlundh LM Orsud HS Olanda MA . Microbiota composition effect on immunotherapy outcomes in colorectal cancer patients: a systematic review. PloS One. (2024) 19:e0307639. doi: 10.1371/journal.pone.0307639 39047017 PMC11268651

[107] El Hajj J Reddy S Verma N Huang EH Kazmi SM . Immune checkpoint inhibitors in pMMR/MSS colorectal cancer. J Gastrointest Cancer. (2023) 54:1017–30. doi: 10.1007/s12029-023-00927-2 37009977 PMC13200515

[108] Van Dingenen L Segers C Wouters S Mysara M Leys N Kumar-Singh S . Dissecting the role of the gut microbiome and fecal microbiota transplantation in radio- and immunotherapy treatment of colorectal cancer. Front Cell Infect Microbiol. (2023) 13:1298264. doi: 10.3389/fcimb.2023.1298264 38035338 PMC10687483

[109] Davar D Dzutsev AK McCulloch JA Rodrigues RR Chauvin J-M Morrison RM . Fecal microbiota transplant overcomes resistance to anti–PD-1 therapy in melanoma patients. Science. (2021) 371:595–602. doi: 10.1126/science.abf3363 33542131 PMC8097968

[110] Baruch EN Youngster I Ben-Betzalel G Ortenberg R Lahat A Katz L . Fecal microbiota transplant promotes response in immunotherapy-refractory melanoma patients. Science. (2021) 371:602–9. doi: 10.1126/science.abb5920 33303685

[111] Duttagupta S Messaoudene M Hunter S Desilets A Jamal R Mihalcioiu C . Fecal microbiota transplantation plus immunotherapy in non-small cell lung cancer and melanoma: the phase 2 FMT-LUMINate trial. Nat Med. (2026) 32:1337–50. doi: 10.1038/s41591-025-04186-5 41606121 PMC13099432

[112] Wong CC Yu J . Gut microbiota in colorectal cancer development and therapy. Nat Rev Clin Oncol. (2023) 20:429–52. doi: 10.1038/s41571-023-00766-x 37169888

[113] Yue B Gao R Wang Z Dou W . Microbiota-host-irinotecan axis: a new insight toward irinotecan chemotherapy. Front Cell Infect Microbiol. (2021) 11:710945. doi: 10.3389/fcimb.2021.710945 34722328 PMC8553258

[114] Wallace BD Roberts AB Pollet RM Ingle JD Biernat KA Pellock SJ . Structure and inhibition of microbiome β-glucuronidases essential to the alleviation of cancer drug toxicity. Chem Biol. (2015) 22:1238–49. doi: 10.1016/j.chembiol.2015.08.005 26364932 PMC4575908

[115] Wallace BD Wang H Lane KT Scott JE Orans J Koo JS . Alleviating cancer drug toxicity by inhibiting a bacterial enzyme. Science. (2010) 330:831–5. doi: 10.1126/science.1191175 21051639 PMC3110694

[116] Funamizu N Lacy CR Fujita K Furukawa K Misawa T Yanaga K . Tetrahydrouridine inhibits cell proliferation through cell cycle regulation regardless of cytidine deaminase expression levels. PloS One. (2012) 7:e37424. doi: 10.1371/journal.pone.0037424 22616006 PMC3353937

[117] Moriconi WJ Slavik M Taylor S . 3-deazauridine (NSC 126849): an interesting modulator of biochemical response. Invest New Drugs. (1986) 4:67–84. doi: 10.1007/BF00172020 2422137

[118] Yuan L Zhang S Li H Yang F Mushtaq N Ullah S . The influence of gut microbiota dysbiosis to the efficacy of 5-fluorouracil treatment on colorectal cancer. BioMed Pharmacother. (2018) 108:184–93. doi: 10.1016/j.biopha.2018.08.165 30219675

[119] Kushwaha M Dalal N Chaudhary S Ahmed A Makharia GK Singh AK . Colorectal cancer biofilm composition reveals distinct bacterial species signature. Appl Microbiol Biotechnol. (2025) 109:159. doi: 10.1007/s00253-025-13537-8 40603621 PMC12222442

[120] Pleguezuelos-Manzano C Puschhof J Rosendahl Huber A van Hoeck A Wood HM Nomburg J . Mutational signature in colorectal cancer caused by genotoxic pks+ E. coli. Nature. (2020) 580:269–73. doi: 10.1038/s41586-020-2080-8 32106218 PMC8142898

[121] Zhou T Ando T Kudo A Sato M Miyoshi N Mutoh M . Screening method toward ClbP-specific inhibitors. Genes Environ. (2023) 45:8. doi: 10.1186/s41021-023-00264-7 36797758 PMC9933310

[122] Xie W Zhong Y-S Li X-J Kang Y-K Peng Q-Y Ying H-Z . Postbiotics in colorectal cancer: intervention mechanisms and perspectives. Front Microbiol. (2024) 15:1360225. doi: 10.3389/fmicb.2024.1360225 38450163 PMC10914944

[123] Vrzáčková N Ruml T Zelenka J . Postbiotics, metabolic signaling, and cancer. Molecules. (2021) 26:1528. doi: 10.3390/molecules26061528 33799580 PMC8000401

[124] Donohoe DR Garge N Zhang X Sun W O’Connell TM Bunger MK . The microbiome and butyrate regulate energy metabolism and autophagy in the mammalian colon. Cell Metab. (2011) 13:517–26. doi: 10.1016/j.cmet.2011.02.018 21531334 PMC3099420

[125] Donohoe DR Collins LB Wali A Bigler R Sun W Bultman SJ . The Warburg effect dictates the mechanism of butyrate-mediated histone acetylation and cell proliferation. Mol Cell. (2012) 48:612–26. doi: 10.1016/j.molcel.2012.08.033 23063526 PMC3513569

[126] Mager LF Burkhard R Pett N Cooke NCA Brown K Ramay H . Microbiome-derived inosine modulates response to checkpoint inhibitor immunotherapy. Science. (2020) 369:1481–9. doi: 10.1126/science.abc3421 32792462

[127] Ma Y Qian X Yu Q Dong Y Wang J Liu H . Inosine prevents colorectal cancer progression by inducing M1 phenotypic polarization of macrophages. Molecules. (2024) 30:123. doi: 10.3390/molecules30010123 39795180 PMC11721193

[128] Konishi H Fujiya M Tanaka H Ueno N Moriichi K Sasajima J . Probiotic-derived ferrichrome inhibits colon cancer progression via JNK-mediated apoptosis. Nat Commun. (2016) 7:12365. doi: 10.1038/ncomms12365 27507542 PMC4987524

[129] Bell HN Rebernick RJ Goyert J Singhal R Kuljanin M Kerk SA . Reuterin in the healthy gut microbiome suppresses colorectal cancer growth through altering redox balance. Cancer Cell. (2022) 40:185–200.e6. doi: 10.1016/j.ccell.2021.12.001 34951957 PMC8847337

[130] Nugrahani AW Hertiani T Haniastuti T Zai K . Anacardic acid as a promising natural antimicrobial agent: mechanisms of action, biofilm inhibition, and advances in nano-encapsulation for enhanced therapeutic efficacy. Fitoterapia. (2025) 187:106951. doi: 10.1016/j.fitote.2025.106951 41135754

[131] Lam KC Araya RE Huang A Chen Q Di Modica M Rodrigues RR . Microbiota triggers STING-type I IFN-dependent monocyte reprogramming of the tumor microenvironment. Cell. (2021) 184:5338–5356.e21. doi: 10.1016/j.cell.2021.09.019 34624222 PMC8650838

[132] Recharla N Geesala R Shi X-Z . Gut microbial metabolite butyrate and its therapeutic role in inflammatory bowel disease: a literature review. Nutrients. (2023) 15:2275. doi: 10.3390/nu15102275 37242159 PMC10221771

[133] Zhang Y Tao Y Gu Y Ma Q . Butyrate facilitates immune clearance of colorectal cancer cells by suppressing STAT1-mediated PD-L1 expression. Clinics. (2023) 78:100303. doi: 10.1016/j.clinsp.2023.100303 37931529 PMC10654141

[134] Li Q Hu W Liu W-X Zhao L-Y Huang D Liu X-D . Streptococcus thermophilus inhibits colorectal tumorigenesis through secreting β-galactosidase. Gastroenterology. (2021) 160:1179–1193.e14. doi: 10.1053/j.gastro.2020.09.003 32920015

[135] Frankel AE Coughlin LA Kim J Froehlich TW Xie Y Frenkel EP . Metagenomic shotgun sequencing and unbiased metabolomic profiling identify specific human gut microbiota and metabolites associated with immune checkpoint therapy efficacy in melanoma patients. Neoplasia. (2017) 19:848–55. doi: 10.1016/j.neo.2017.08.004 28923537 PMC5602478

[136] Kim SY . Immunotherapy for microsatellite-stable colorectal cancer: overcoming resistance and exploring novel therapeutic strategies. Ann Coloproctology. (2026) 42:47–57. doi: 10.3393/ac.2025.01354.0193 41802306 PMC12971173

[137] Di J Liu J Zhang X . Mechanisms underlying immunotherapy resistance in microsatellite-stable colorectal cancer. Oncol Adv. (2025) 3:e00021. doi: 10.14218/OnA.2025.00021

[138] Gao Y Bi D Xie R Li M Guo J Liu H . Fusobacterium nucleatum enhances the efficacy of PD-L1 blockade in colorectal cancer. Signal Transduct Target Ther. (2021) 6:398. doi: 10.1038/s41392-021-00795-x 34795206 PMC8602417

[139] Zhang L Jiang L Yu L Li Q Tian X He J . Inhibition of UBA6 by inosine augments tumour immunogenicity and responses. Nat Commun. (2022) 13:5413. doi: 10.1038/s41467-022-33116-z 36109526 PMC9478149

[140] Fluckiger A Daillère R Sassi M Sixt BS Liu P Loos F . Cross-reactivity between tumor MHC class I–restricted antigens and an enterococcal bacteriophage. Science. (2020) 369:936–42. doi: 10.1126/science.aax0701 32820119

[141] Bessell CA Isser A Havel JJ Lee S Bell DR Hickey JW . Commensal bacteria stimulate antitumor responses via T cell cross-reactivity. JCI Insight. (2020) 5:e135597. doi: 10.1172/jci.insight.135597 32324171 PMC7205429

[142] Carneiro W Michelon I Simões A Taha K Cavalcante L . 1134 outcomes of fecal microbiota transplantation (FMT) in cancer patients receiving immunotherapy: a systematic review and meta-analysis. Regular Young Investigator Award Abstracts. (2025) 13:A1301–2. doi: 10.1136/jitc-2025-SITC2025.1134

[143] Fernandes R Jabbarizadeh B Rajeh A Hong MMY Baines KJ Ernst S . Fecal microbiota transplantation plus immunotherapy in metastatic renal cell carcinoma: the phase 1 PERFORM trial. Nat Med. (2026) 32:1325–36. doi: 10.1038/s41591-025-04183-8 41606120 PMC13099417

[144] Porcari S Ciccarese C Heidrich V Rondinella D Quaranta G Severino A . Fecal microbiota transplantation plus pembrolizumab and axitinib in metastatic renal cell carcinoma: the randomized phase 2 TACITO trial. Nat Med. (2026) 32:1316–24. doi: 10.1038/s41591-025-04189-2 41606119 PMC13099650

[145] De Clercq NC Van Den Ende T Prodan A Hemke R Davids M Pedersen HK . Fecal microbiota transplantation from overweight or obese donors in cachectic patients with advanced gastroesophageal cancer: a randomized, double-blind, placebo-controlled, phase II study. Clin Cancer Res. (2021) 27:3784–92. doi: 10.1158/1078-0432.CCR-20-4918 33883174

[146] U.S. Food and Drug Administration . Fecal Microbiota for Transplantation: Safety Communication- Risk of Serious Adverse Reactions Due to Transmission of Multi-Drug Resistant Organisms (2019). Available online at: https://www.fda.gov/safety/medical-product-safety-information/fecal-microbiota-transplantation-safety-communication-risk-serious-adverse-reactions-due (Accessed July 20, 2026).

[147] Vedanta Biosciences, Inc . Phase 1 study of VE800 and nivolumab in patients with selected types of advanced or metastatic cancer Clinicaltrials.Gov (2022). Available online at: https://clinicaltrials.gov/study/NCT04208958 (Accessed May 1, 2026).

[148] Dizman N Meza L Bergerot P Alcantara M Dorff T Lyou Y . Nivolumab plus ipilimumab with or without live bacterial supplementation in metastatic renal cell carcinoma: a randomized phase 1 trial. Nat Med. (2022) 28:704–12. doi: 10.1038/s41591-022-01694-6 35228755 PMC9018425

[149] Spencer C McQuade J Gopalakrishnan V McCulloch J Vetizou M Cogdill A . Dietary fiber and probiotics influence the gut microbiome and melanoma immunotherapy response. Science. (2021) 374:1632–40. doi: 10.1126/science.aaz7015 34941392 PMC8970537

[150] Zhang S-L Mao Y-Q Zhang Z-Y Li Z-M Kong C-Y Chen H-L . Pectin supplement significantly enhanced the anti-PD-1 efficacy in tumor-bearing mice humanized with gut microbiota from patients with colorectal cancer. Theranostics. (2021) 11:4155–70. doi: 10.7150/thno.54476 33754054 PMC7977465

[151] Han K Nam J Xu J Sun X Huang X Animasahun O . Generation of systemic antitumour immunity via the in situ modulation of the gut microbiome by an orally administered inulin gel. Nat BioMed Eng. (2021) 5:1377–88. doi: 10.1038/s41551-021-00749-2 34168321 PMC8595497

[152] Wang J-W Hsu W-H Yu F-J Kuo F-C Liu C-J Kuo C-H . *Clostridium butyricum MIYAIRI 588* reduces colorectal adenomatous polyp recurrence: a randomized crossover trial. Oncol Res. (2025) 33:3907–22. doi: 10.32604/or.2025.070432 41425719 PMC12712693

[153] Yang Y Guo Y Ding Y Li J Liang L Shi D . Perioperative administration of CBM588 in colorectal cancer radical surgery: a single-center, randomized controlled trial. Cell Rep Med. (2025) 6:102234. doi: 10.1016/j.xcrm.2025.102234 40633539 PMC12281423

[154] Brusnic O Onisor D Boicean A Hasegan A Ichim C Guzun A . Fecal microbiota transplantation: insights into colon carcinogenesis and immune regulation. J Clin Med. (2024) 13:6578. doi: 10.3390/jcm13216578 39518717 PMC11547077

[155] Yu H Li X-X Han X Chen B-X Zhang X-H Gao S . Fecal microbiota transplantation inhibits colorectal cancer progression: reversing intestinal microbial dysbiosis to enhance anti-cancer immune responses. Front Microbiol. (2023) 14:1126808. doi: 10.3389/fmicb.2023.1126808 37143538 PMC10151806

[156] Zheng D-W Dong X Pan P Chen K-W Fan J-X Cheng S-X . Phage-guided modulation of the gut microbiota of mouse models of colorectal cancer augments their responses to chemotherapy. Nat BioMed Eng. (2019) 3:717–28. doi: 10.1038/s41551-019-0423-2 31332342

[157] Leventhal D Sokolovska A Li N Plescia C Kolodziej S Gallant C . Immunotherapy with engineered bacteria by targeting the STING pathway for anti-tumor immunity. Nat Commun. (2020) 11:2739. doi: 10.1038/s41467-020-16602-0 32483165 PMC7264239

[158] Canale F Basso C Antonini G Perotti M Li N Sokolovska A . Metabolic modulation of tumours with engineered bacteria for immunotherapy. Nature. (2021) 598:662–6. doi: 10.1038/s41586-021-04003-2 34616044

[159] Zhu Y Yin Y Li Y Wang Q Chen Y Gu K . Bibliometric analysis of genetically engineered bacteria in tumor therapy. Front Microbiol. (2026) 17:1794032. doi: 10.3389/fmicb.2026.1794032 42094765 PMC13141310

[160] Gurbatri C Lia I Vincent R Coker C Castro S Treuting P . Engineered probiotics for local tumor delivery of checkpoint blockade nanobodies. Sci Transl Med. (2020) 12:eaax0876. doi: 10.1126/scitranslmed.aax0876 32051224 PMC7685004

[161] Lei X Yu Y Chung C Qiu Z Zhang Y Chu T . Oral immunization with *Listeria monocytogenes* vaccine enhances immunotherapy for protective immunity in murine models of colorectal cancer. J Immunother Cancer. (2026) 14:e011570. doi: 10.1136/jitc-2025-011570 41644269 PMC12878199

[162] Cao Y Wang J Wang H Sun X Fang F Li S . Targeting *Peptostreptococcus anaerobius* with an iron‐based nanozyme reverses ferroptosis resistance and enhances antitumor immunity in colorectal cancer. Adv Sci. (2026) 13:e16272. doi: 10.1002/advs.202516272 41524179 PMC13292267

[163] Khan A Mujahid A Tareen A Amin A Khan S Hadi D . Next-generation immune cell therapies for lung cancer: advances in CAR-T, NK, and TIL strategies. Front Med. (2026) 13:1787086. doi: 10.3389/fmed.2026.1787086 41859142 PMC12995752

[164] Tareen A Zehra N Arsalan S Ahmed A Khan A Moosajee M . The influence of immune checkpoint inhibitors on treatment results in cancer patients: real-world evidence from a single institution in a low-middle-income country. J Clin Oncol. (2026) 44:e23370. doi: 10.1200/JCO.2026.44.16_suppl.e2337042852411

[165] Galeano Niño J Wu H LaCourse K Kempchinsky A Baryiames A Barber B . Effect of the intratumoral microbiota on spatial and cellular heterogeneity in cancer. Nature. (2022) 611:810–7. doi: 10.1038/s41586-022-05435-0 36385528 PMC9684076

[166] Ramesh A Subbarayan R Shrestha R Adtani P . Exploring fecal microbiota transplantation: potential benefits, associated risks, and challenges in cancer treatment. Cancer Rep. (2026) 9:e70455. doi: 10.1002/cnr2.70455 41496455 PMC12775360

